# Rope making in the Aurignacian of Central Europe more than 35,000 years ago

**DOI:** 10.1126/sciadv.adh5217

**Published:** 2024-01-31

**Authors:** Nicholas J. Conard, Veerle Rots

**Affiliations:** ^1^ Department of Early Prehistory and Quaternary Ecology, Schloss Hohentübingen, University of Tübingen, 72070 Tübingen, Germany.; ^2^ Senckenberg Centre for Human Evolution and Paleoenvironment, Schloss Hohentübingen, University of Tübingen, 72070 Tübingen, Germany.; ^3^ F.R.S.-FNRS, TraceoLab, University of Liège, 4000 Liège, Belgium.

## Abstract

Evidence for the manufacture and use of fiber technology such as rope and twine is rare in the Paleolithic, despite the widely held view that such artifacts were in regular use during the Pleistocene. On the basis of the discovery of a more than 35,000-year-old perforated baton made from mammoth ivory at Hohle Fels Cave in Ach Valley of southwestern Germany together with experimental studies, we are now able to demonstrate one way people of the early Upper Paleolithic manufactured rope. This work contributes to our understanding of the evolution of technology, cooperative work, and Paleolithic social organization.

## Teaser

A more than 35,000-year-old mammoth ivory tool provides insights into how early Upper Paleolithic people made rope.

## INTRODUCTION

Paleolithic life is difficult to imagine without technology to tie materials together for a myriad of purposes (*1*, *2*). Until now, the best evidence for string, twine, and rope came from impressions in clay, from depictions in Paleolithic art (*3*, *4*), and from traces of fibers that may originate from these materials (*5*, *6*). Reconstructing technology for working fibers and making rope and textiles requires a combination of empirical finds from excavations and experimental programs that test how artifacts may have been used. Here, we report the discovery of artifacts and experimental and technofunctional studies that provide evidence for how some early Upper Paleolithic people made rope. We also consider some of the socioeconomic implications of rope making as an early form of cooperative production of material culture.

## RESULTS

**Fig. 1.**
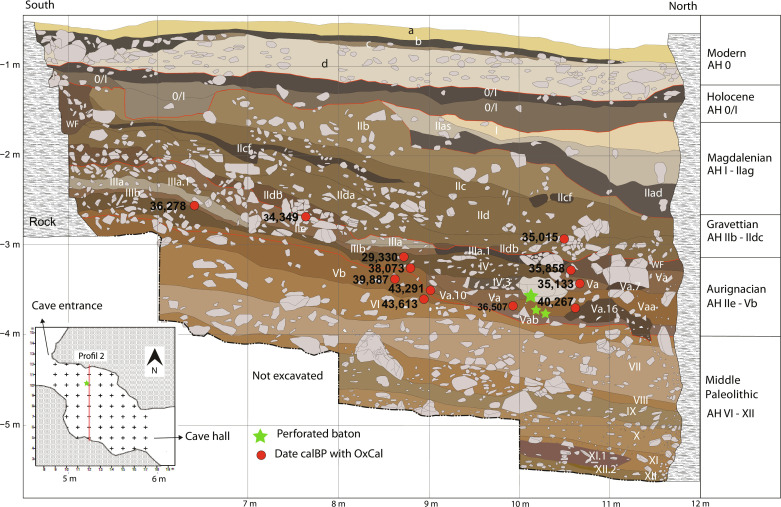
North-south stratigraphic Profile 2 at Hohle Fels. The large green star shows the position of the 13 fragments of the perforated baton that were recovered in place. The small green stars show the approximate position of the two fragments found during waterscreening. The red circles show the location of radiocarbon dates from anthropogenically modified bones within 50 cm east and west of Profile 2. (Image: A. Janas, University of Tübingen.)

**Fig. 2.**
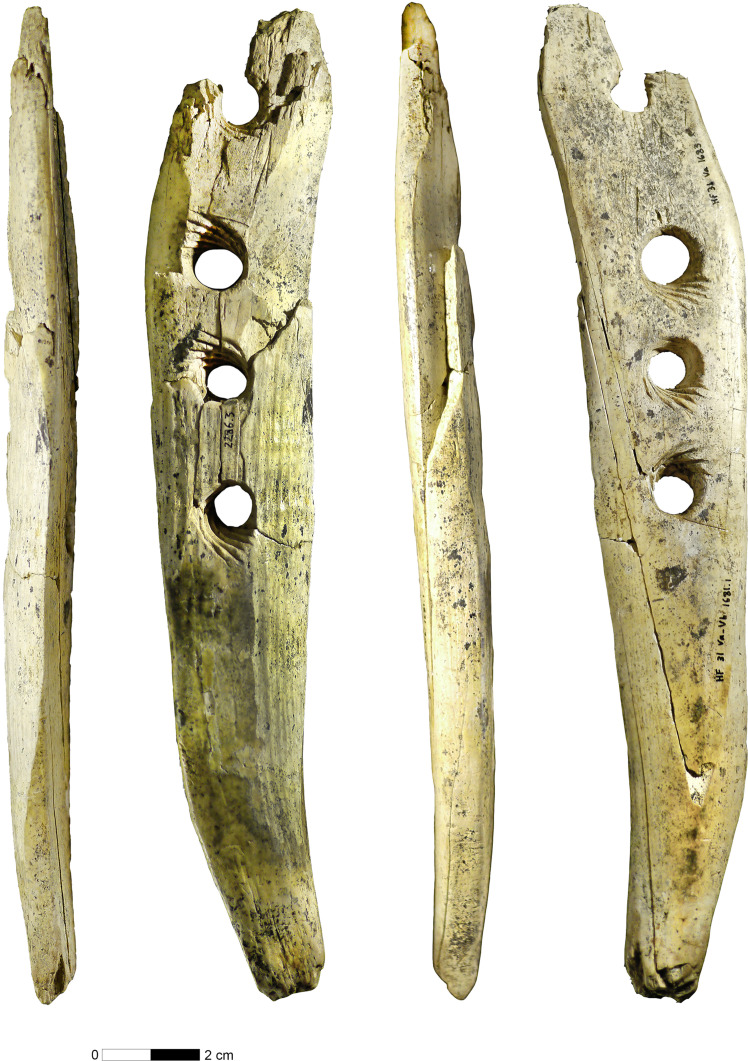
Ivory perforated baton from Hohle Fels Cave, southwestern Germany with four views. Convex surface second from the left, flat surface far right. Distal end up, handle down. (Photo: H. Jensen, University of Tübingen.)

Excavators at Hohle Fels Cave in the Ach Valley of the Swabian Jura of southwestern Germany found 13 pieces of worked mammoth ivory in archaeological horizon AH Va on 15 August 2015 (text S1) (*7*). The team recovered two additional fragments of the same artifact during waterscreening sediments from profile cleaning of AH Va and Vb and from profile collapse containing sediments from AH Va, Vaa, and Vb (Fig. 1). Because all of the pieces found in situ originated from AH Va, we argue that the Aurignacian occupants of the cave discarded the artifact during the formation of AH Va and not during the formation of the underlying strata. The AH Va complex is rich in Aurignacian artifacts and has been dated with radiocarbon to between 35,000 and 40,000 years ago (*8*). The pieces of worked mammoth ivory could be refitted into a well-preserved and nearly complete perforated baton, with four holes containing precisely carved spiral grooves (Fig. 2 and section S2). The artifact is 20.4 cm long, 3.6 cm wide, 1.5 cm thick, and the four holes vary slightly in size and in nature of the rifling. The surface of the tool is well preserved, and the edges of the six grooves within each hole are sharp, suggesting that the artifact had not experienced heavy use. Here, we present this remarkable artifact and the results of comprehensive use-wear and residue analyses combined with experimental studies to determine its function.

Despite considerable investment in radiocarbon dating, micromorphology, and taphonomic analyses (*9*), the exact age of the perforated baton cannot be determined with a high degree of certainty. Figure 1 presents the stratigraphic position of the artifact, which was recovered within the north-south Profile 2 that transects the site and represents one of the major stratigraphic reference profiles at Hohle Fels. The figure also depicts all radiocarbon dates currently available from anthropogenically modifeid bones that fall within 50 cm east or west of the profile. The radiocarbon dates from AH Va fall in the range from 35,000 and 40,000 years ago, with only one of these dates falling older than 36.5 thousand calibrated years before the present (ka cal BP). The high variability in the dates likely originates in part from the diverse factors that can affect the reliability of measuring radiocarbon in faunal samples as well as interlaboratory comparability. The geological and taphonomic setting of the horizons also contributes to the broad spread of the radiocarbon signal.

The Aurignacian strata at Hohle Fels have a slope downward from the inside of the cave toward the entrance of between 5° and 10°. As numerous studies have shown (*9*), the mode of sedimentation is largely colluvial movement from the sediment cone in the main hall of the cave with a minor eolian contribution entering though the entrance of the cave. The bulk of the silt and clay in the matrix of the cave deposits entered the site through a chimney in the rear of cave, with additional sediments coming from weathered limestone and material entering the cave through small cracks in the ceiling. Equally important, Hohle Fels experienced intense occupation by cave bears over many tens of thousands of years and substantial human occupation during many phases of the Middle and Upper Paleolithic. Cave bears routinely hibernated and denned in the cave, and people spent large amounts of time in the cave, especially in the colder months. Both bears and hominins regularly moved around inside the cave and moved in and out of the cave while digging and modifying the deposits and facilitating down-slope movement of sediments. Thus, it comes as no surprise that some of the strata in the cave contain finds that yield a wide range of ages (*9*, *10*).

In an attempt to establish better chronological control over this part of the sequence, we dated 19 anthropogenically modified faunal remains from AH Va and AH Vaa (Table 1). The 7 median ages from AH Vaa are usually older than the 12 median ages from AH Va, with the dates from AH Vaa complex falling between 34.8 and 40.2 ka cal BP, while the dates from AH Va complex fall between 35.1 and 39.0 ka cal BP. Given that the perforated baton was recovered at the base of AH Va, the series of radiocarbon dates does little to provide a precise date of the artifact. We can, however, be confident that the perforated baton is no younger than 35 ka BP and postdates 40 ka BP, with the age probably closer to the younger part of this spectrum. Without dating the artifact itself, we are unable to provide a more specific age for the new find.

**Table 1.** Radiocarbon dates on anthropogenically modified bones from archaeological horizons at Hohle Fels. Calibration (*45*, *46*).

Laboratory ID	AH	GH	Quadrant	Find ID	Uncalibrated age	Uncertainty	Cal BP/OxCal 95.4% probability	Median cal BP OxCal	First publication	Description	Comment
KIA 16038*	IIIa	6a	78	2161	29,840	210	34,681– 33,953	34,349	(*36*)	Reindeer, femur	
OxA-4601*	IIIa	6a	28	796	30,550	550	36,149–34,139	35,015	(*47*)	Bone	
OxA-33485*	IIIb	6b	87	270	31,900	450	37,320–35,325	36,278	This paper	Mammoth/wooly rhino, rib	
KIA 3504*	IV	7	79	2475	25,020	360	30,053–28,627	29,330	This paper	Horse, rib	
OxA-31618*	IV.3	7	28	1467	31,500	400	36,812–35,105	35,858	This paper	Ibex, tibia	
OxA-24152*	Va.9	7a	28	1425	30,760	370	35,978–34,486	35,133	This paper	Horse, rib	
OxA-31616	Va.7	7a	11	476	31,190	400	36,305–34,705	35,589	This paper	Reindeer, metatarsus	
OxA-31623	Va.10	7a	30	1094	31,500	400	36,812–33,105	35,858	This paper	Horse, mandible	
OxA-31561	Va.10	7a	30	1043	31,500	400	36,812–33,105	35,858	This paper	Horse, rib	
OxA-31559	Va.10	7a	99	2364	31,600	450	37,016–35,096	35,957	This paper	Horse, femur	
KIA 35464	Va	7a	10	946	31,750	260	36,645–35,457	36,094	(*16*)	Horse, tibia/radius	
OxA-19783	Va.10	7a	30	1078	31,760	200	36,519–35,560	36,114	(*48*)	Reindeer, tibia	
KIA 35463	Va	7a	24	1564	32,030	280	37,021–35,776	36,392	(*16*)	Horse, rib	Dated twice
KIA 35462	Va	7a	10	1019	32,090	350	37,275–35,647	36,499	(*16*)	Reindeer, vertebra	
KIA 35460	Va	7a	24	1474	32,370	280	37,403–36,170	36,706	(*16*)	Mammoth, vertebra	
KIA 35459	Va	7a	24	1604	32,550	300	37,730–36,186	36,904	(*16*)	Horse, radius	
OxA-24151	Va	7a	24	1564	34,050	550	40,476–37,471	39,049	This paper	Horse, rib	Dated twice
ETH-42292	Vaa	7aa	11	863	30,385	275	35,339–34,345	34,815	This paper	Mammoth, rib	
OxA-24153	Vaa	7aa	25	1133	32,400	450	38,469–35,845	36,834	This paper	Horse, rib	
OxA-31624	Vaa.11	7aa	25	1227	33,250	500	39,375–36,675	38,042	This paper	Reindeer, metatarsus	
OxA-X-2418-34	Vaa	7aa	11	863	33,350	550	39,562–36,680	38,157	This paper	Mammoth, rib	Comment lab: sample produced 4.3 mg of collagen, below the 5-mg threshold
OxA-X-2418-35	Vaa.12	7aa	11	872	34,000	650	40,549–37,173	38,941	This paper	Horse, femur	Comment lab: sample produced 4.3 mg of collagen, below the 5-mg threshold
OxA-19859	Va.10	7a	89	1632	34,570	260	40,389–39,252	39,726	(*48*)	Mammoth/wooly rhino, rib	
OxA-31560*	Vaa.16	7aa	28	2012	35,100	650	41,565–39,110	40,267	This paper	Horse, undetermined long bone fragment	
OxA-19782*	Vb	8	30	1276	32,140	310	37,240–35,849	36,507	(*48*)	Horse, hyoid	
KIA 16035*	Vb	8	79	2670	33,290	270	37,141–35,213	38,073	(*48*)	Horse, undetermined long bone fragment	
OxA-19779*	Vb	8	89	1705	34,720	280	40,531–39,378	39,887	(*48*)	Horse, tibia	
OxA-19781*	Vb	8	89	1614	40,000	500	44,207–42,654	43,291	(*48*)	Ibex, tibia	
OxA-31621*	VI	9	89	1804	40,100	1,200	45,515–42,242	43,613	This paper	Horse,humerus	

*Present in Fig. 1.

In addition to the artifact from Hohle Fels, four other perforated batons have been recovered in Aurignacian deposits of the Swabian Caves (Table 2). In 1983, excavators at Geißenklösterle Cave in the Ach Valley 2 km downstream from Hohle Fels recovered a nearly identical object composed of approximately 30 weathered fragments of mammoth ivory adjacent to a hearth. Most of the fragments originated from AH IIb, with fewer pieces from IIa (*11*, *12*). In addition to the four grooved holes, one edge of this find bears a series of notches. The perforated baton from Geißenklösterle, like other finds of this kind, was considered by J. Hahn, the head of the excavation, and other researchers to be some kind of artwork rather than a tool (*12*, *13*). Dates for the Aurignacian of Geißenklösterle fall between 42.5 and 37 ka BP, with ages from the upper Aurignacian of the AH II complex typically falling in the later half of this range (*14*).

**Table 2.** Aurignacian perforated batons from the Swabian Jura.

Site	Baton	AH	Cultural group	Material	Number of perforations	Excavated	First publication
Hohle Fels	1	Va	Lower Aurignacian	Ivory	4	2015	(*7*)
Geißenklösterle	1	II (a + b)	Upper Aurignacian	Ivory	4	1983	(*11*, *12*)
Vogelherd “gorget“	1	V	Aurignacian	Ivory	2	1931	(*15*)
Vogelherd “bullroarer“	2	V	Aurignacian	Ivory	1	1931	(*15*)
Vogelherd	3	IV	Aurignacian	Ivory	1	1931	(*15*)

In 1931, archaeologists at Vogelherd in the Lone Valley recovered three perforated batons made from mammoth ivory. One specimen from the rich Aurignacian layer AH V bears two holes with spiral grooves. In keeping with the prevailing views of the day, G. Riek, the excavation director, interpreted the find to be a gorget and a symbolic artifact (*15*). Riek interpreted a second perforated baton from AH V with a single grooved hole to be a bullroarer (*15*). A third broken perforated baton from Aurignacian layer AH IV bears a hole with grooves and markings that extend to its lateral edges. Because of the lack of piece-plotting in 1931, the exact provenience of the finds from Riek’s excavation is not known. Nonetheless, the radiocarbon dates for the Aurignacian of Vogelherd fall in the same range as the Aurignacian of neighboring sites of the Ach Valley (*16*).

Although perforated batons, referred to in German as *Lochstab* (pl. *Lochstäbe*) and in French as *bâton percé*, are well known from all of the major periods of the Upper Paleolithic, four-holed, perforated batons of the kind found at Hohle Fels and Geißenklösterle are not known in other regions, and given their early Aurignacian age, these artifacts count among the earliest perforated batons known.

Most perforated batons are made from reindeer antler, and although examples with multiple holes are known, they typically have a single hole (*17*). Perforated batons have been documented in the Aurignacian and Gravettian periods dating in Central Europe to before the Last Glacial Maximum (LGM), but they are particularly numerous during the Magdalenian, which postdates the LGM (*18*, *19*). In general, the insides of the holes are smooth, but some specimens include spiral grooves inside or adjacent to the holes similar to that seen on the ivory artifacts from the Swabian Aurignacian (*20*–*28*). Some researchers have suggested that the grooves may originate from reworking the holes rather than their serving a function in their own right (*19*). Many interpretations have viewed these finds as symbols of power, ritual objects that are sometimes associated with burials or artworks of various kinds (*13*,*29*–*31*). The fact that many of these finds were carefully engraved and decorated with markings strengthened the tradition of viewing them as being artistic objects primarily associated with symbolic or ritual beliefs. Researchers viewed the spiral grooves, when present, as symbolic markings rather than as functional features. Perforated batons have also been hypothesized to be shaft straighteners or to be tools used for leather working (*31*).

The discovery of the perforated ivory artifact from Hohle Fels motivated us to reexamine the find from Geißenklösterle and to consider the hypothesis that these carefully made artifacts severed as tools rather than as symbolic artifacts. We suspected that the holes with spiral grooves were made to have something fed through them, which led us to hypothesize that the artifact may have served to align fibers to make rope or twine. While twine can be made by hand without tools, by pulling and twisting plant fibers together (*32*), we tested whether or not the perforated baton from Hohle Fels could have been used to align, twist, and combine fibers to make rope. We also examined how Paleolithic people could make such a precision tool and looked for use-wear and residues with the goal of determining how the object had been used.

**Fig. 3.**
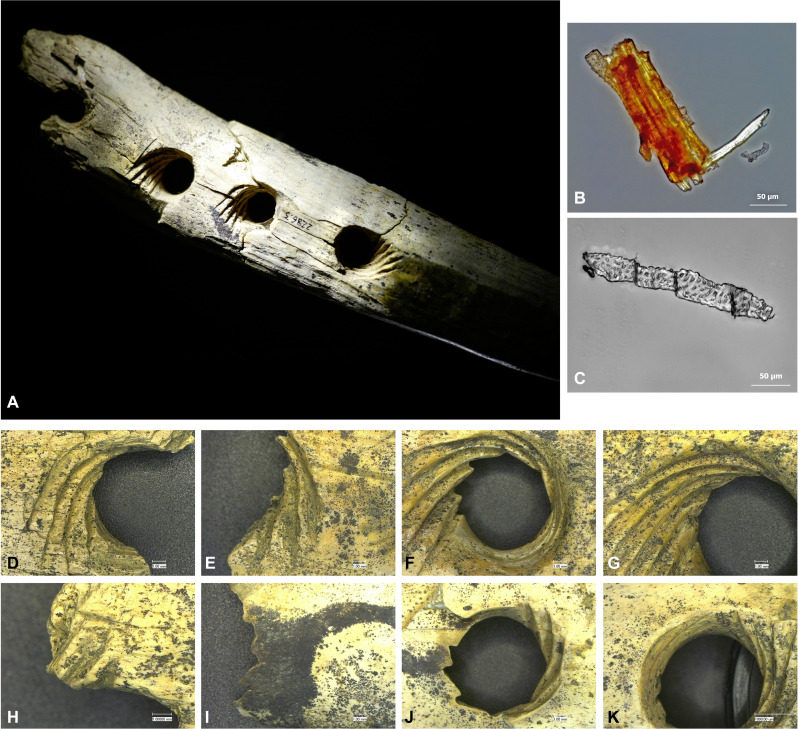
Macro- and microscopic images of the ivory perforated baton from Hohle Fels and residue evidence. (**A**) Ivory perforated baton. (**B**) Plant tissue extracted from Lochstab (transmitted-light microscopy, POL, ×400). (**C**) Possible tracheid extracted from Lochstab (transmitted-light microscopy, POL, ×400). (**D** to **K**) Details of the grooved holes according to their position on the artifact. Images are taken on the main fragmented parts of the Lochstab before refitting. Both faces of each hole are depicted in the order as depicted in (A) [distal hole: (D) and (H), note fracture in groove on (H); left central hole: (E) and (I); right central hole: (F) and (J); proximal hole/near handle: (G) and (K)]. [Photo: (A): H. Jensen, University of Tübingen; (B) and (C): D. Cnuts, University of Liège; (D) to (K); V. Rots, University of Liège.]

The* Lochstab* is made from a large piece of split ivory and preserves a convex and a flat surface. Part of the convex surface is formed by the bedding plane separating the dentine and the cementum. The flat side of the artifact is cut through the dentine, and the handle of the tool is located toward the distal end of the tusk. We examined the *Lochstab* microscopically to determine how it was manufactured (text S3). The four holes are biconical in section and were perforated from both faces of the artifact. Their perfect alignment suggest that the maker of the artifact first drilled the holes, after which the holes were enlarged from both sides. The holes are nearly perfect circles with the two central holes slightly closer to each other than they are to the more lateral holes. A smaller hole of 7 mm diameter alternates with a larger hole of 9 mm diameter. Independent of their size, all holes preserve uniform, incised grooves at regular distances with a smooth rotation (Fig. 3). The maker of the tool incised the grooves from each of the faces of the artifact. The grooves align perfectly in the center of the hole. While the grooves of the two central holes cover a full circle of 360°, the grooves of the outer two holes cover a semicircle of 180°. The grooves rotate clockwise when viewed from the convex surface. While the grooves of the central holes start toward the distal end of the tool on the convex surface, the grooves of the outer two holes start toward the handle. The fracture of the most distal hole occurred within a groove; its cause is uncertain and could have happened during manufacture, use, or postdepositionally.

Grooves are V-shaped and were manufactured using a sharp stone edge. The insides of the holes are smooth, but striations from their manufacture are still visible. We observed no signs of wear, rounding, or abrasion other than those linked to production. Aside from the holes and grooves, no additional intentional incisions or grooves could be documented. Incorporated within some remaining sediment, we observed vegetal fibers within the grooves.

The ivory perforated baton from Geisenklösterle is housed in the State Museum in Stuttgart (text S4). Its state of preservation is poor with clear deterioration as a result of taphonomic agents and postdepositional weathering. The grooves are similar to the artifact from Hohle Fels with clearly intentional and V-shaped profiles, but in this case, the grooves are heavily worn, suggesting intensive use in an abrasive activity. The lateral edge of the ivory artifact shows cuts that were intentionally made with a sharp edged tool as indicated by their clear V-shape profiles. These incisions have widened and rounded through use and subsequent taphonomic alteration. While the grooves within the holes are similar in both specimens, the incisions on the lateral edge of the perforated baton from Geißenklösterle are not present on the artifact from Hohle Fels. This represents a difference between both tools, or it could be the result of the artifact from Hohle Fels having received little or no use.

After excavation, the perforated baton from Hohle Fels was carefully cleaned and slowly dried to avoid cracking and fragmentation, to which ivory is prone. Microscopic examination revealed fibers adhering in the area of the holes and grooves (Fig. 3 and text S3). We examined the extractions taken from the small samples of sediment collected from the grooves and from the surface of the tool for residues. A predominance of plant fibers could be noted, some of which occurred in clusters and were associated with plant tissue. Plant fibers may easily come from the environment. Therefore, we compared the residues from the perforated baton to the residues found within the sediment from the area around the artifact. These sediment samples revealed few plant fibers, suggesting that the concentration found on the artifact is linked to the manufacturing and/or functional context of the piece. We also examined six stone artifacts found in association with the *Lochstab* to evaluate whether the tool could have broken during manufacturing or early on in its use. Four lithic artifacts revealed informative plant fibers; one of these artifacts also preserved plant tissue. In two cases, the plant fibers were accompanied by hard animal residues. At least one of the lithic artifacts revealed use-wear related to perforating hard animal material like ivory. A fifth tool revealed use-wear related to cutting hard animal material but no animal residues. A final tool revealed no traces or residues.

On the basis of the morphology of the perforated baton from Hohle Fels, the residues, and wear traces on associated stone artifacts and on the Geissenklösterle *Lochstab*, we hypothesized that these finds served as rope making tools. To test this hypothesis, we reproduced the *Lochstab* and used it for fiber processing and rope manufacture. We performed experiments to test the functionality of individual holes and of the four holes in combination with sinew from deer, flax (*Linum*), hemp (*Cannabis*), cattail (*Typha*), linden (*Tilia*), willow (*Salix*), and nettles (*Urtica*) (text S5).

Individual holes of the *Lochstab* did not prove effective for pretreating sinew, flax, nettles, and hemp, but we achieved positive results for cattail, linden, and willow. Cattail was particularly applicable because the *Lochstab* could help to remove the starch for consumption by crushing the outer harder surface of the stems while separating the fibers for cordage. The use of cattail for making rope is well documented ethnographically, and archaeological accounts exist, in particular for later periods (*33*). Cattail is highly useful for food, cordage, and basketry (*33*, *34*). Pollen has not yet been studied in the Aurignacian deposits; however, pollen of the *Typha*/*Sparganium* type has been documented in the overlying Gravettian layers at Hohle Fels, and there is every reason to expect that cattails would have grown in protected, low-lying spots in the Ach Valley during the generally milder Aurignacian period. Although thermophilic linden is not present in the botanical record of Hohle Fels, willow is abundant among the piece-plotted finds of wood charcoal from most layers including the Aurignacian horizons (*35*) from which excavators recovered the* Lochstab*.

Turning to the use of multiple holes in combination to make rope, we focused our tests mainly on cattail. We found that the* Lochstab* was not essential for manufacturing thin rope, which can be done manually. The tool’s relevance lies in making thicker, stronger rope consisting of two to four strands. We twisted and fed bundles of cattail leaves through the holes. The holes help to maintain a regular thickness of the strands and facilitate the addition of new material necessary for making long stretches of rope. The grooves help to break down the leaves and orient the fibers while maintaining the torsion needed for rope making. The four-holed tool is then pulled with regular speed over the strands (text S6). Behind the tool, the strands combine automatically into a rope as a result of their twisting tension. The number of holes used determines the thickness of the rope. Because one person is needed to twist and maintain tension on each of the strands and one to operate the *Lochstab*, three to five people would be needed to use a four-holed *Lochstab* for rope making. Our experiments using cattail and four or five participants typically produced 5 m of strong and supple rope in 10 min (text S5).

## DISCUSSION

The discovery of what is likely an Aurignacian rope making tool at Hohle Fels helps to answer the question of how Upper Paleolithic people produced rope. Just as every camper, hunter, or collector today knows that rope and twine are useful for countless practical tasks, Paleolithic life would be difficult without rope, twine, or leather straps of some kind. Every ethnographically studied group of hunters and gatherers uses rope or twine for a wide variety of purposes (*32*). The cooperative work required to make rope using the perforated batons from the Swabian Aurignacian required complex and well-coordinated communication and shared goals. This kind of activity would have underlined the reciprocal relationships that bound group members together and gave them a competitive advantage over groups who had less well developed community activities with delayed profits from investment of technology and labor. We can now add rope making technology using perforated batons to the innovations associated with the arrival of Aurignacian hunter-gatherers in Central Europe (*36*, *37*).

## MATERIALS AND METHODS

After careful excavation and initial cleaning, the ivory *Lochstab* found at Hohle Fels was examined for residues and wear traces. In addition, the stone artifacts found in the same find squares were screened and six artifacts with possible signs of use were selected for a more detailed study (text S3). We examined the *Lochstab* in the Material Culture Laboratory at the University of Tübingen with the aid of a Keyence microscope and an AxioLab A1 microscope (×50 to ×500). Residue extractions made using scalpel and pipette were exported to the University of Liège for further study. Sediment samples taken during excavation of the area surrounding the ivory piece were transported to Liège for closer examination. We screened the lithic artifacts at the University of Tübingen with the aid of a stereoscopic microscope. We transported selected artifacts to the University of Liège for closer examination. Analysis was performed with a Zeiss V20 stereoscopic microscope (×12 to ×240) and with an Olympus BX51M metallurgical microscope (×50 to ×1000). Extracted residues were examined with a Zeiss Axioscope transmitted-light microscope (×50 to ×1000) and with a JEOL scanning electron microscope–energy-dispersive x-ray spectroscope. Detailed results of the microscopic wear and residue analyses are included in text S3.

The ivory *Lochstab* from Geissenklösterle is housed in the State Museum in Stuttgart. The find could be examined by V.R. in September 2016. The artifact proved to be in a poor state of preservation with clear deterioration as a result of taphonomic agents (text S4 and figs. S1 and S2). Given its very fragile nature, the artifact had to be handled with extreme care, but it was nevertheless examined under low magnification as best as possible. Results are included in text S4.

An explorative experimental program was performed to test the functionality of the artifact in fiber processing and rope manufacturing and to evaluate its possible relevance in related activities (text S5). We devoted special attention to evaluating whether the grooves within the holes could have a functional role. Numerous accounts exist on what raw materials are good sources of fibers and on how to make ropes (*38*–*44*). Rope and twine are easily made by hand without any tools by rolling the fibers between the palm of the hand and the thigh, but in our experiment, we focused specifically on the manufacture of sturdier ropes, which are more difficult to produce by hand. We evaluated how the *Lochstab* could be integrated in the manufacturing process and to what extent the process benefits from its use. These results are included in text S5.

## Supplementary Materials

### This PDF file includes:

Texts S1 to S5 Figs. S1 to S16 Table S1 Legend for movie S1 References
