# Supplementary material for: Rope making in the Aurignacian of Central Europe more than 35,000 years ago
Source: Sci Adv. 2024 Jan 31;10(5):eadh5217. doi: 10.1126/sciadv.adh5217 (PMC10830101; doi:10.1126/sciadv.adh5217)
Supplement: Supplementary file 1 — Texts S1 to S5 Figs. S1 to S16 Table S1 Legend for movie S1 References [file sciadv.adh5217_sm.pdf]

Science Advances
AAAS

Supplementary Materials for

Rope making in the Aurignacian of Central Europe more than 35,000 years ago

Nicholas J. Conard and Veerle Rots

Corresponding author: Nicholas J. Conard, nicholas.conard@uni-tuebingen.de

Sci. Adv. **10**, eadh5217 (2024)
DOI: 10.1126/sciadv.adh5217

The PDF file includes:

Texts S1 to S5
Figs. S1 to S16
Table S1
Legend for movie S1
References

Other Supplementary Material for this manuscript includes the following:

Movie S1

Supplementary Text 1:

Find context of the Lochstab

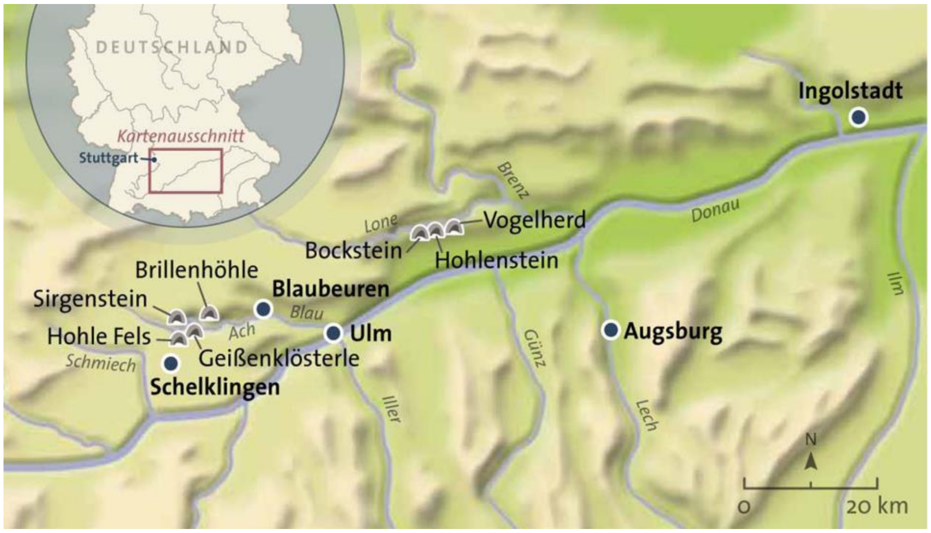

Fig. S1. Location Hohle Fels and other sites mentioned in the text.

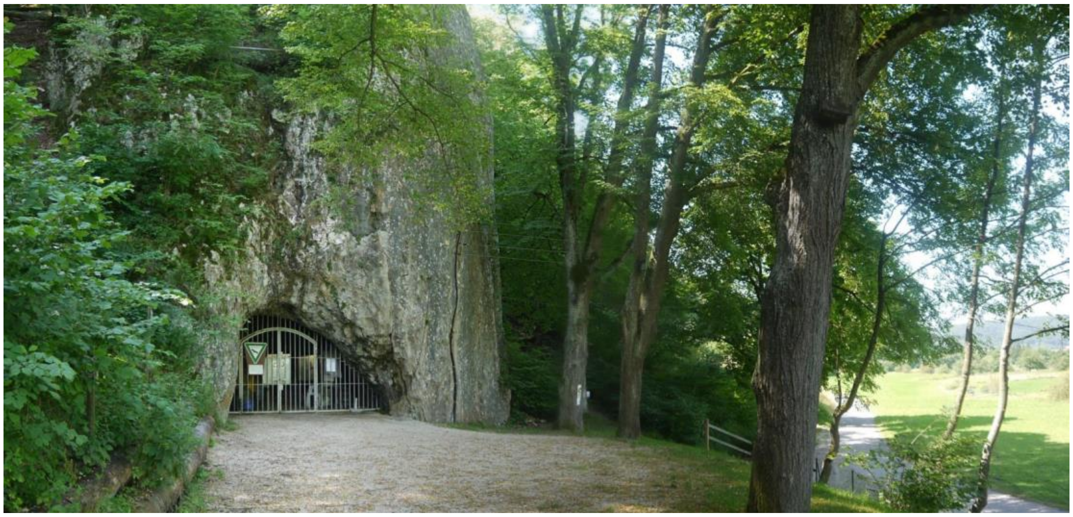

Fig. S2. View of the entrance of Hohle Fels Cave overlooking the Ach Valley.

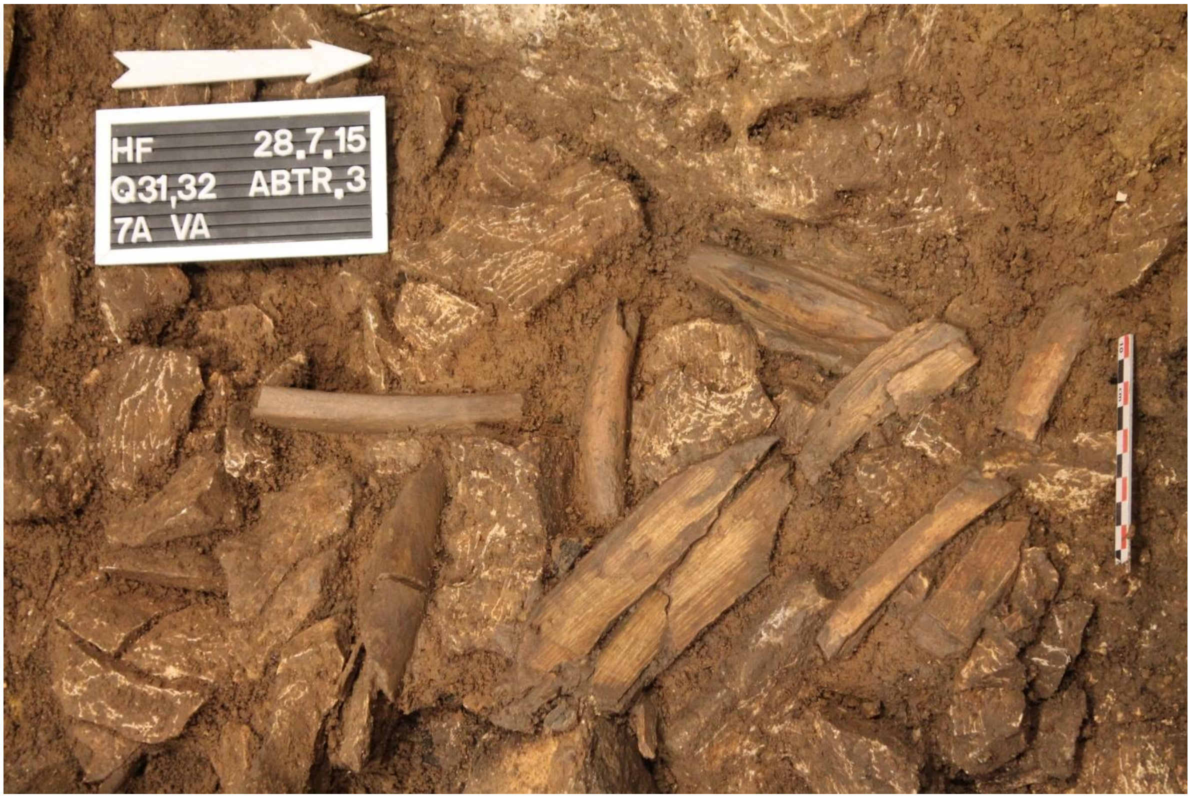

Fig. S3. Hohle Fels. Concentration of worked pieces of mammoth ivory from Aurignacian layer AH Va. Photo: University of Tübingen.

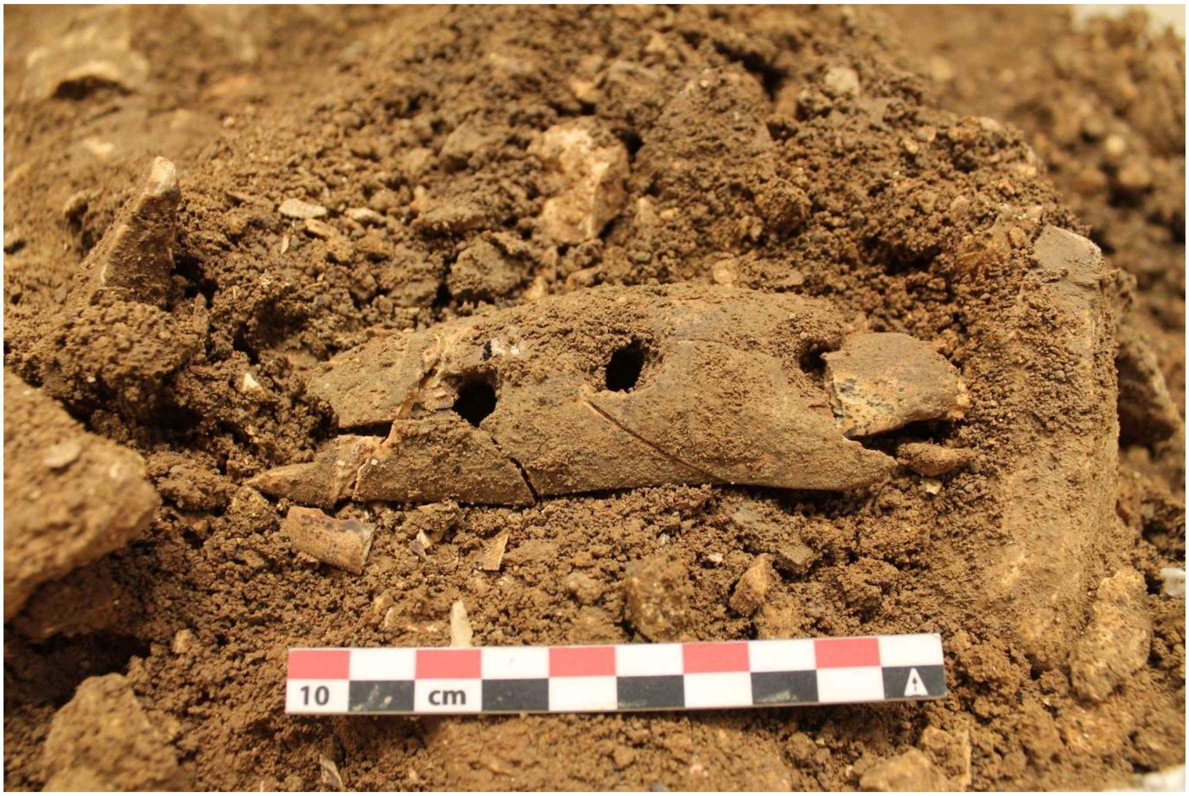

Fig. S4. Hohle Fels. Perforated baton of mammoth ivory from Aurignacian layer AH Va at the time of discovery in July 2015. Photo: University of Tübingen.

Supplementary Text 2:

3D model of the Lochstab

The 3D model was deposited on Dryad and can be downloaded here: doi:10.5061/dryad.xd2547dqb

Supplementary Text 3:

Microscopic analysis of residues and wear traces

Microscopic analysis of the *Lochstab*

A low magnification use-wear analysis was performed on the different fragments of the ivory *Lochstab*, and no obvious signs of wear, rounding or abrasion were observed aside from what is expected from the manufacturing process itself. The insides of the holes are smooth but still clearly show the striations related to the manufacturing process. No explicit use-related smoothing is visible. The grooves appear fresh and do not show any distinct signs of use or abrasion. The grooves are v-shaped and were clearly manufactured by using a sharp stone edge. Aside from the holes and grooves, no further signs of explicit intentional incisions or grooves were observed on the artifact. Some adhering vegetal fibres were observed within the grooves of the holes, incorporated within some remaining sediment.

More attention was subsequently devoted to all possible residues through low and high magnification analysis. After excavation, the *Lochstab* had been carefully cleaned of adhering sediment and slowly dried in order to prevent cracking and fragmentation. While this process is essential, it limits the possibilities of the residue analysis with extractions that could only be performed on the remaining minor sample of sediment that was collected from within the grooves of the tool and from some surface areas.

The limited sediment that was still preserved in some of the grooves of the central two holes and on the top and lateral surface of the ivory *Lochstab* was carefully extracted with a clean scalpel. No pipet or ultra-sonic bath extractions were performed in order not to damage the artifact. Six scalpel extractions were made: two from sediment preserved in the grooves (two central holes) (1683/1 & 1683/2), two from the surface around the holes (1683/3 & 1683/4), one from a recent surface near the hole (1683/5), and one from the lateral edge of one of the extremities (location of the broken hole) (1683/6). The residue content of these sediment samples was examined. In total, it contains two wood fragments, one tracheid, 50 plant fibres (cellulose) and a cluster of plant fibres, seven plant tissues, one hair fibre, over 1200 starch grains, four ivory fragments, and two root fragments (see Table S1).

	Square unit	Layer	ANIMAL				VEGETAL							Iron oxide	Unidentified
			Hard matter	Ivory	Hide	Hair fiber	Plant fiber	Cluster of plant fibers	Tracheid	Plant tissue	Wood	Starch grain	Root		
Lochstab															
HF/1683/1	31	7a		1			4		1		1 1		1		
HF/1683/2				1			14			3			1		
HF/1683/3				1			18								
HF/1683/4							8								
HF/1683/5				1			2			3		~100			
HF/1683/6						1	5	1				~1200			
Sediment samples															
HF/1013	31	7a/BPA/Va					6								
HF/1574	32	7a/BPD/Va		6			3			2					
HF/1286	11	10/BPA/VII					2								
HF/1823	25	10/BPA/VII		1			8								
HF/1014	32	7a/BPA/Va					10				1				
HF/1573	31	7a/BPA/Va		26			2				1				
Stone artefacts															
Perforator (ID 1725)	31	7a/Va	1				21					~50		1	2
Burin spall (ID 1698)	31	7a/Va	6		1	1	39				2	~20			
Small blade fragment (ID 1526)	31	7a/Va					24								1
Distal fragment partially crested bladelet (ID 2069)	28	7aa/Vaa					14			3	1	~100			1
Retouched blade fragment (ID 1435)	31	7a/Va					13								
Small flake (ID 1545)	31	7a/Va					5								

Table S1. Residues identified on the *Lochstab*, within the surrounding sediment and on associated stone artifacts (grey: uncertain identifications; yellow: moderately certain; green: certain identifications).

While the rootlets are obviously due to taphonomy, also cellulose / plant fibres should be treated with caution as they are all-round in the environment, in particular on living sites, and they may thus also be deposited accidentally on the artifact *(49)*, which questions interpretation of fibre processing and rope making based on a single fibre on a single tool (cf. *6*). Ideally, plant fibres should only be considered as potentially indicative of use when associated with plant tissue. Considering that the artifact was cleaned, the fibres at least adhered strongly to the *Lochstab* (Fig S5). While the plant fibres may come from the sediment, their frequency is high given the small sample involved (only 6 slides could be made based on the amount of extracted sediment). Given this important concentration of fibres, a direct link with use has to be considered as well, in particular given that plant tissue was also observed. The fibre morphology proved insufficiently characteristic to allow a more detailed identification of the species. The cluster of plant fibres (Figure S5a) is particularly interesting and likely related to use. The wood fragments lack internal structure and their identification was therefore only based on colour, their amorphous morphology, sharp edges, and the absence of birefringence.

The ivory fragments (Figure S5c+d) observed within the extractions obviously come from the piece itself, i.e. loose particles that were removed while the adhering sediment was extracted. One definite hair was observed (Figure S5b). Its section at the base is quite small (9.47 µm) and much smaller than human hair (including eyebrows or eyelashes) according to published data (*50* mention a thickness between 40-120 µm for human scalp hair), therefore, the hair is likely animal. It is similar to at least some of the hairs that were documented on the stone tools of the Aurignacian levels by Bruce Hardy *(51)*, in particular the illustrated one that was tentatively attributed to mustelids (*51*: Figure 2). Given that only one hair was observed, it is likely to have accumulated on the piece as a result of residues present in the general use environment. However, the hair was found in the single extraction that could be made from the distal lateral edges and other hairs may have been washed off through the essential cleaning immediately after the discovery of the ivory piece. Whether or not the hair is deteriorated is difficult to evaluate (*52*), but the original strong adherence of the hair to the ivory piece contradicts recent contamination.

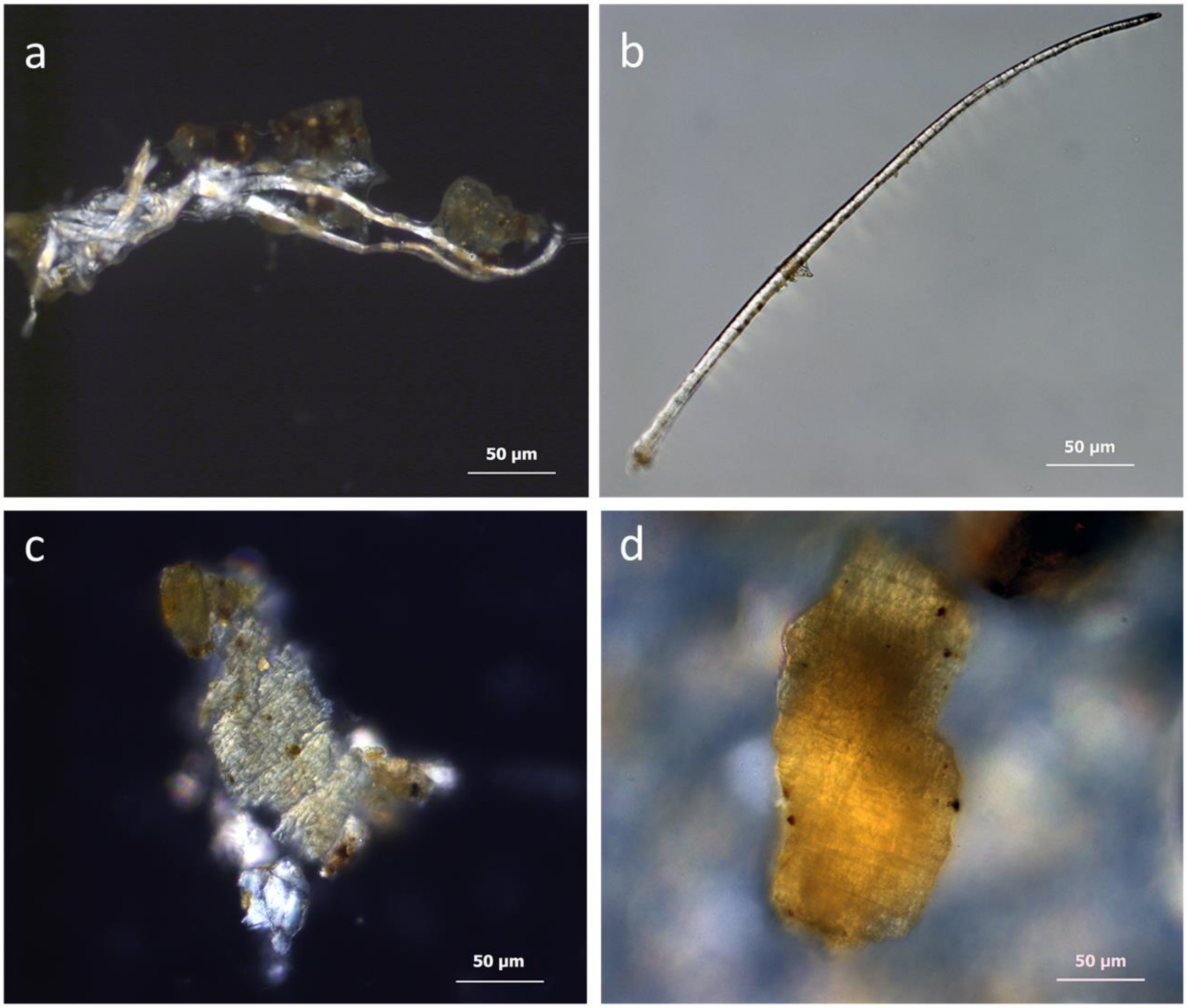

Figure S5. Residues extracted from the ivory Lochstab and observed with transmitted light microscopy: a) Cluster of plant fibres (POL; 400x); b) Isolated hair (POL; 400x); c) possible ivory fragment (POL; 400x); d) possible ivory fragment (POL; 400x). Photos: D. Cnuts, University of Liège.

For evaluating the cause of deposition of the residues, it is important to examine what was observed per extraction zone on the *Lochstab*. The rootlets were found in the sediment from the grooves, as well as the wood fragments and part of the plant fibres. The remaining sediment that could be sampled from the surface of the artifact serves as a kind of reference of what is likely due to taphonomy. Only some plant fibres were found in this extraction, confirming the likelihood that minimally part of the fibres are purely taphonomic. No plant tissue was observed in that extraction, which further corroborates that the combined occurrence of both plant fibres and tissue is a reliable indicator for a use-related origin. The extractions from the surface around the hole also showed that specific combination, including longer fibres. The cluster of plant fibres as well as the hair was found in the extraction on the lateral edge of the piece on the extremity. No tissue was associated.

Microscopic analysis of sediment samples

Sediment samples were screened to contextualize the residues found on the artifact and to evaluate whether the plant fibres and the other residues were a standard component of the surrounding sediment and could thus have been deposited on the *Lochstab* as a result of taphonomic processes. Six sediment samples were processed in total: two sediment samples derive from the same square as the ivory artifact, two from an adjacent square, and two samples derive from a non-anthropogenic level. Plant cellulose fibres were found in all sediment samples, also the non-anthropogenic ones, confirming that these plant fibres indeed need to be considered with caution. Cellulose is everywhere in the environment and only very diagnostic fibres or high frequencies should be considered in the context of tool use. On a total of 9 slides, 31 plant fibres were counted, implying that their frequency is much lower than what was observed on the ivory piece (see Table S1). Ivory fragments were abundant in the sediment samples from the anthropogenic squares, but they were absent from the non-anthropogenic squares. While ivory fragments for square 32 were infrequent, small and not very diagnostic, the ivory fragments from the square in which the ivory piece was found, square 31, were abundant, large and very explicit, especially in one of the samples (nr 1573). Amongst the fragments, also a clear tiny ivory flake (chip) was identified. It could derive from on-site ivory production or from the defragmentation of the ivory piece with some dispersal of microscopically-sized fragments in the surrounding sediment. For some slides of square 31, a frequency of about 25 ivory fragments was calculated, which is very high. Plant tissue was observed in one sediment sample only, 1574 (Figure S6a), derived from an adjacent square, but it was not associated with fibres.

The residue content corresponds broadly to what was also recovered from the ivory piece, suggesting that these residues were part of the use setting. Ivory production in the square is suggested by various larger fragments, while also plant / fibre processing seems to have taken place. Even though plant fibres are part of the sediment, their frequency is significantly higher on the ivory piece, which is suggestive for a potential link with the actual use of the *Lochstab* in the processing of plant fibres. It was therefore explored whether the *Lochstab* could have functioned in fibre processing and more in particular rope manufacturing through experimentation (see Supplementary Text 5) and through the analysis of associated stone artifacts.

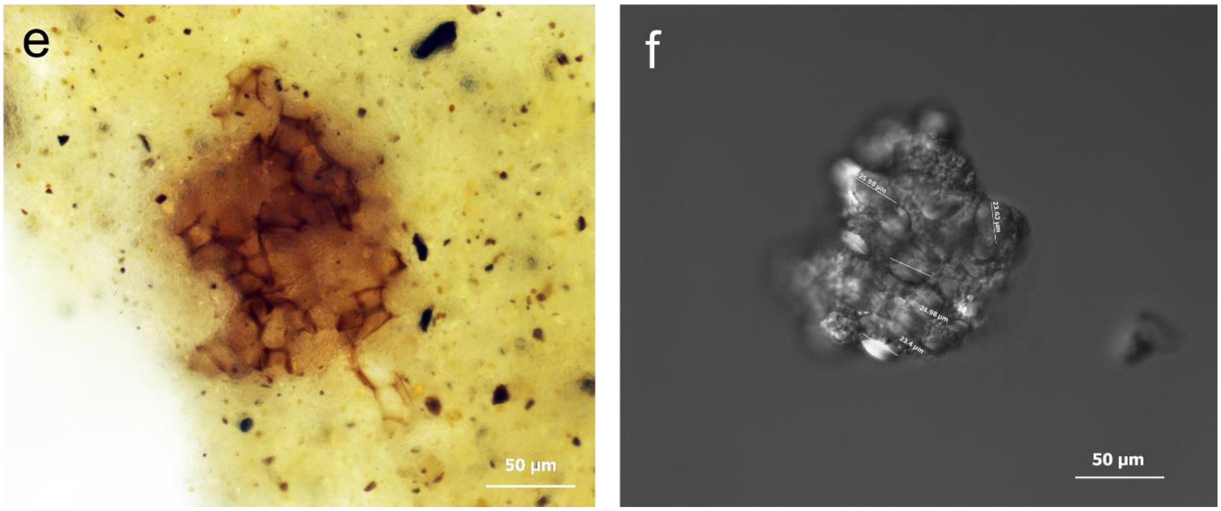

Figure S6. Residues extracted from the sediment or associated stone artifacts and observed with transmitted light microscopy: a) Plant tissue within sediment sample 1574 (POL; 400x); b) starch grains from tool 1725 (POL; 400x). Photos: D. Cnuts, University of Liège.

Microscopic analysis of selected associated stone artifacts

The stone artifacts associated with the ivory artifact were screened for signs of potential use in ivory manufacturing and/or plant processing to clarify the find context of the *Lochstab* on a functional level. If the stone artifacts proved to be used for ivory (hard animal material) processing, it could reinforce the hypothesis that the piece was broken in manufacture or early in its use cycle. If stone artifacts proved to be used in plant working, it could reinforce the hypothesis with regard to the use of the *Lochstab* in plant fibre processing and/or rope manufacture. Attention was mainly focused on the residues to permit contextualization of the residues found on the *Lochstab*, but wear evidence was also examined.

The artifacts found in association with the *Lochstab* were screened and six artifacts (unit 31, one from unit 28), both retouched and unretouched, were selected for closer examination (Figure S7). Five of these were found in the same square as the ivory piece, one was found in another square in the same area. Artifacts were selected on the basis of possible signs of use when viewed under a stereoscopic microscope and a potential relevance for ivory working.

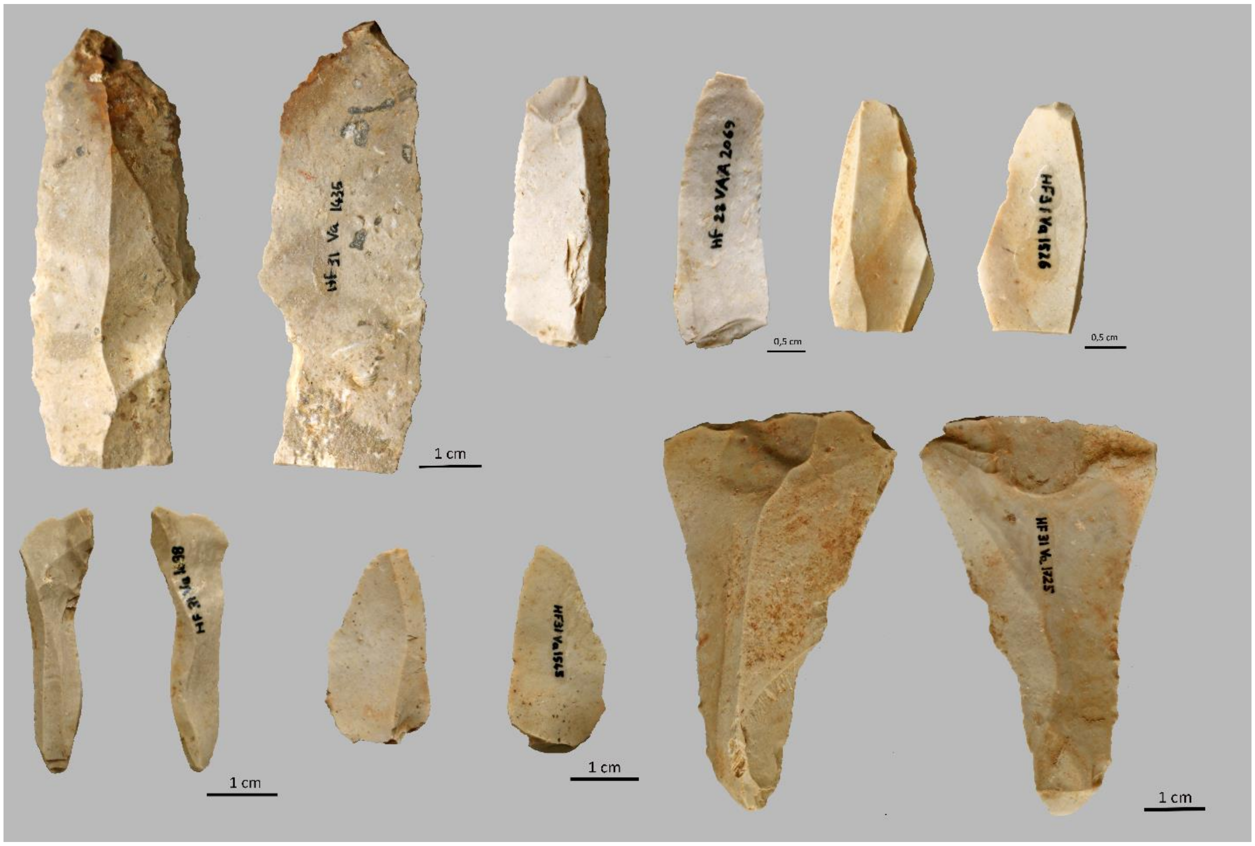

Figure S7. Stone tools examined for residues. First row from left to right: Retouched blade fragment (ID 1435), Partially crested bladelet (ID 2069), Blade fragment (ID 1526). Second row from left to right: Burin spall (ID 1698), Flake (ID 1545), Proximally fractured flake (ID 1725).

Since detailed use-wear analysis with a metallurgical microscope requires cleaning of the artifacts, residue extractions were made first. Localized residue extractions were taken with the aid of a pipet in zones linked with possible use-wear traces as viewed under a stereoscopic microscope. After examination of these area-specific extractions, it was considered relevant to perform an additional residue extraction of the entire artifact with an ultra-sonic bath in order to guarantee a complete view on the residue content. It also facilitates subsequent handling as no further precautions are required.

Two artifacts (ID 1435, ID 1545) only showed cellulose fibres that can be ignored in terms of their use as no other residue types were associated and their frequency remained low. The cellulose fibres therefore need to be attributed to taphonomy or contamination. The small flake (ID 1545) does not show diagnostic use-wear either. The retouched blade fragment (ID 1435) however does show evidence of use-wear on its edge, including poorly developed use polish associated with slight rounding and intense edge damage. The traces are suggestive of a cutting motion on hard animal material. The ventral extremity shows intense rounding associated with polish, which reveals intense friction and compares to what can be expected for prehension traces in the case of a use on hard animal material (see *53*).

Four other artifacts show relevant residues, more in particular plant fibres associated with plant tissue and hard animal matter. The residues are discussed together with the use-wear data.

A proximally fractured flake (ID 1725) with macroscopic damage on the edges and extremities (some of it production-related) shows wear and residues indicative of use. Plant fibres were found next to hard animal residues, a starch cluster (*Figure S3.2b*), iron oxide and unknown residues. The starch is not due to contamination and in combination with the fibres, the residues seem suggestive for plant processing. In terms of the wear traces, little polish is present, but the edge damage appears indicative of a contact with hard animal materials in a perforating motion. Ivory working is thus a possibility.

A burin spall (ID 1698) shows several residues, of which particularly the plant fibres, the hair and the 6 bone fragments are important. These residues are combined with little wear polish and some edge damage, but the evidence is not diagnostic enough to indicate use with certainty.

A small blade fragment (ID 1526) shows a number of plant fibres and unknown residues that seem to derive from a hard material. The residues are not indicative for any use. Little wear traces are visible. There is minor polish formation but it is insufficiently developed to argue a certain link with use; the same goes for the limited edge damage. If the flake was used, working of softer materials like plant are in any case more likely than hard animal materials.

A distal fragment of a partially crested bladelet (ID 2069) is derived from another square, but it provided a reasonable number of plant-related residues including plant tissue and some plant fibres. The large size and the unaltered appearance of the starch grains (i.e. absence of fissures) suggests that they are most likely recent contamination. Use in some form of plant processing is possible but remains uncertain. Little wear evidence could be observed.

The analysis of the stone artifacts does not provide conclusive evidence with regard to the interpretation of the discard state of the *Lochstab* given that both evidence with regard to manufacturing (hard animal material) as to possible fibre processing could be observed.

Conclusion

Plant residues are significantly more frequent on the *Lochstab* and the stone artifacts than in the surrounding sediment. This suggests that the *Lochstab* may indeed have been used – or intended to have been used – in plant processing activities, more in particular fibre processing and rope making. However, the residue analysis did not provide sufficiently convincing evidence in support of this hypothesis. The lack of abundant residues is not an argument against the use of the *Lochstab* because the artifact was well-cleaned after recovery to prevent ivory deterioration. The absence of diagnostic wear within the grooves and the holes of the *Lochstab* from Hohle Fels, in comparison to wear observed on the *Lochstab* of Geissenklösterle (see Supplementary Text 4), leads us to suggest that the Hohle Fels *Lochstab* may have remained unused or little used. Two stone artifacts possibly testify a use on hard animal material, though

it cannot be determined whether this would be ivory as distinguishing between different hard animal materials is complicated and requires well-developed wear. Use of some stone artifacts in plant processing is possible but remains inconclusive in this stage.

Supplementary Text 4:

Examination of the ivory *Lochstab* of Geissenklösterle

The ivory Lochstab from Geissenklösterle has a poor state of preservation with clear deterioration due to taphonomic agents (Fig. S8). Given its very fragile nature, the artefact had to be handled with extreme care, but it was nevertheless examined under low magnification as best as possible.

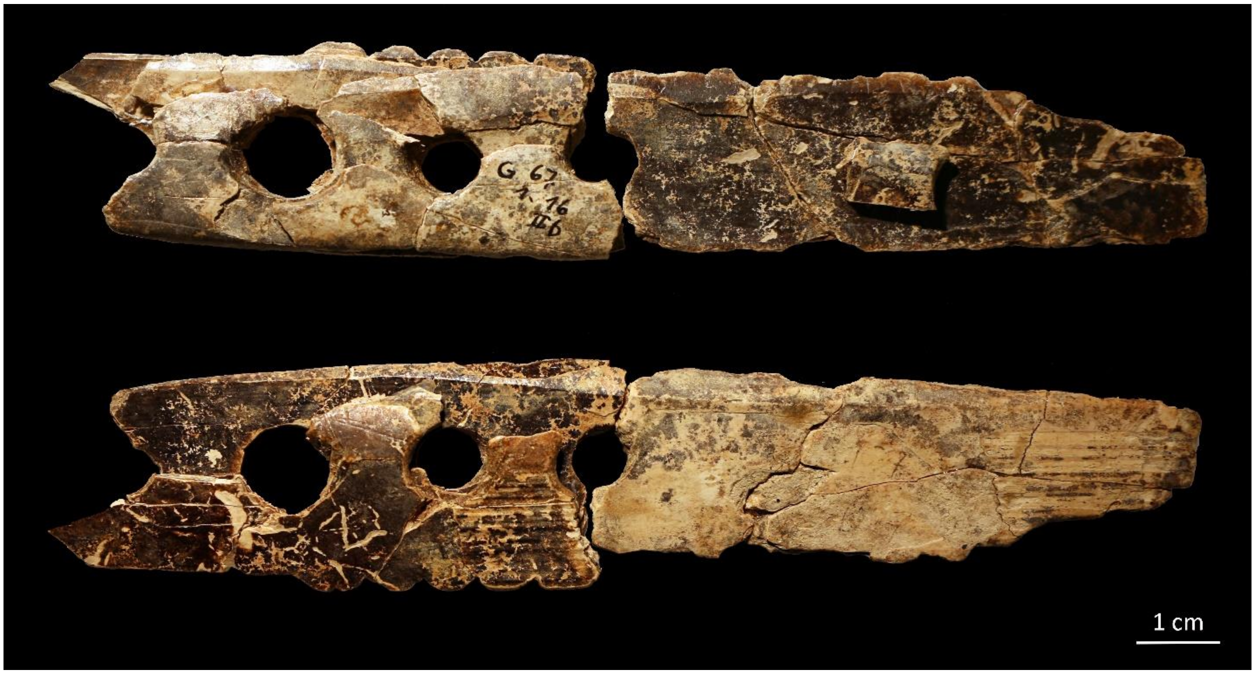

Fig. S8. Macro image of the Lochstab of Geissenklösterle

The Lochstab shows grooves within each of the four holes, similar to the ones on the artifact from Hohle Fels. These grooves are clearly intentional and show a V-shaped profile and they are heavily worn and run down suggesting intensive use in an abrasive activity (Fig. S9). Even though preservation is not advantageous to the examination, all grooves seem to have a 180° rotation and to be identical to the hole situated closest to the handle for the Hohle Fels piece. The grooves within the hole on the broken extremity of the Geissenklösterle piece could not be verified, given the important damage. All grooves on the sufficiently preserved holes are identical to one another in terms of their initiation and termination, which contrasts with the Hohle Fels piece.

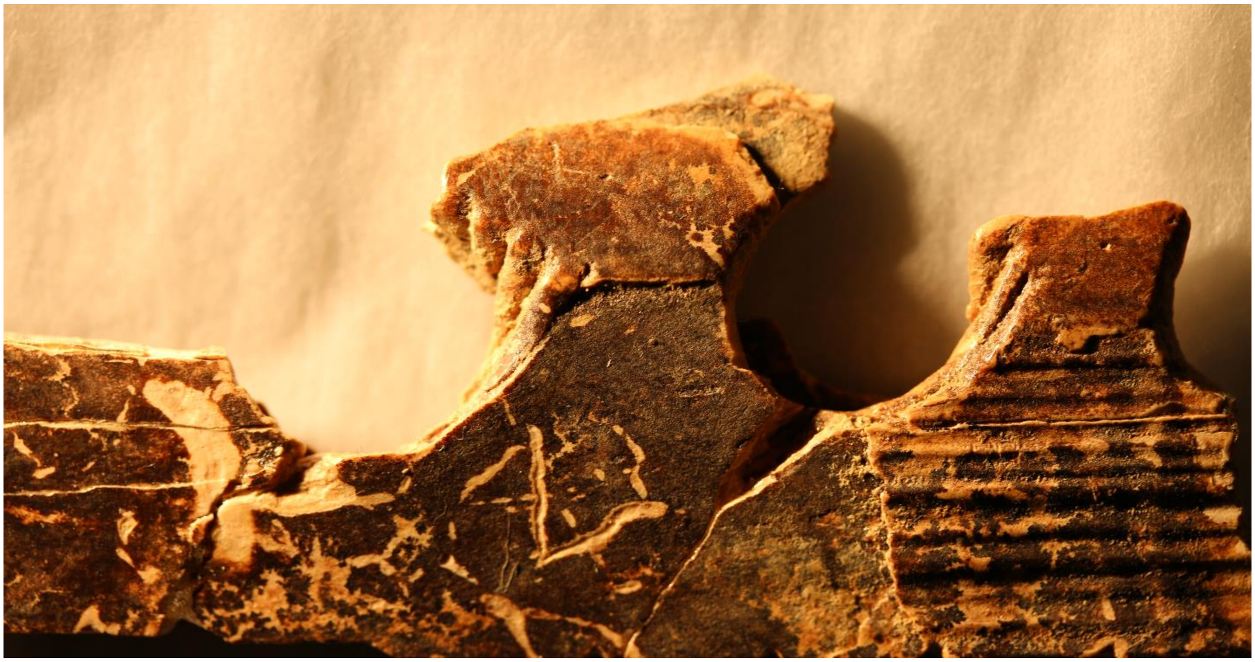

Fig. S9. Detail of the grooves

Interestingly, the fracture on the distal extremity is nearly identical in terms of location and characteristics, which lends support to the fact that this fracture would be functional in nature and linked to the pressure exerted on the piece and the holes during use.

The lateral edge of the ivory Lochstab from Geissenklösterle shows clear indentations, intentionally made with a sharp edge as shown by their explicit V-shape (Fig. S10). These incisions have widened and rounded through use and subsequent taphonomic alteration. Such incisions on the lateral edge of the Lochstab are absent from the find from Hohle Fels. This either represents a difference between both Lochstabe, or it could be the result of the tool from Hohle Fels having received little or no use.

The poor preservation state of the Geissenklösterle piece hindered a more detailed analysis.

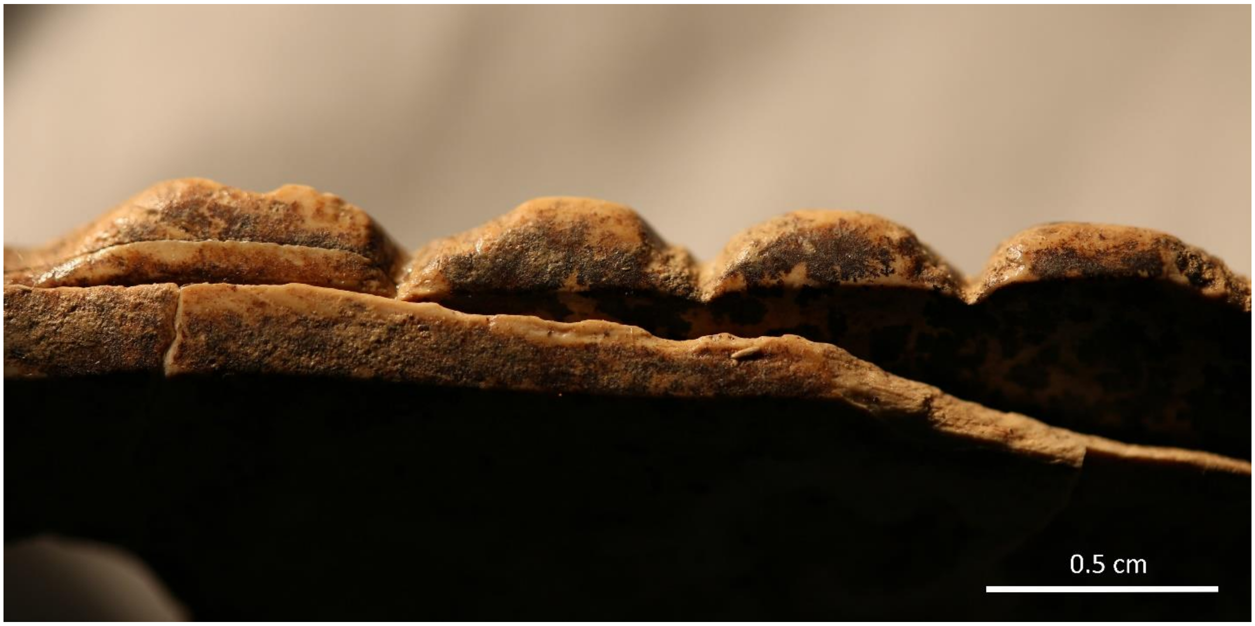

Fig. S10. Indentations on lateral edge

In contrast to the Hohle Fels Lochstabe, there is little doubt about the fact that the piece from Geissenklösterle was intensively used. Significant wear is visible and this wear appears to correspond to what can be expected in the case of fibre working and rope manufacturing. The most distal hole may well have fractured in the process.

Supplementary Text 5:

Experiments in fibre processing and rope manufacturing

Reproduction of the *Lochstab*

Several reproductions of the *Lochstab* were manufactured in both hardwood (buxus) and bone (horse and cattle) by an experienced experimenter, Christian Lepers, University of Liège. Attention was paid to reproduce the artefact as close as possible for what the position of the holes and their widths concerns. Also grooves were incised. As ivory is not readily available, particularly not in the size required here, we used other raw materials in this experiment. First a hardwood example was made, in particular to reflect on the artefact and its production. Incisions were easy to produce in hardwood, but they could not be kept as actual 'grooves' because the wood simply broke off. Subsequently, we used bone to reproduce the Lochstab, despite known differences in hardness (5 for bone versus about 3 for ivory on Moh's scale) and structure in comparison to ivory. Ivory has good elasticity and strength and is a preferred material for carvings. A total of three items were produced in bone, with varying degrees of finishing (Fig. S11). Next, also an example in warthog tooth ivory was made. This artefact reached the highest degree of completion (Fig. S12).

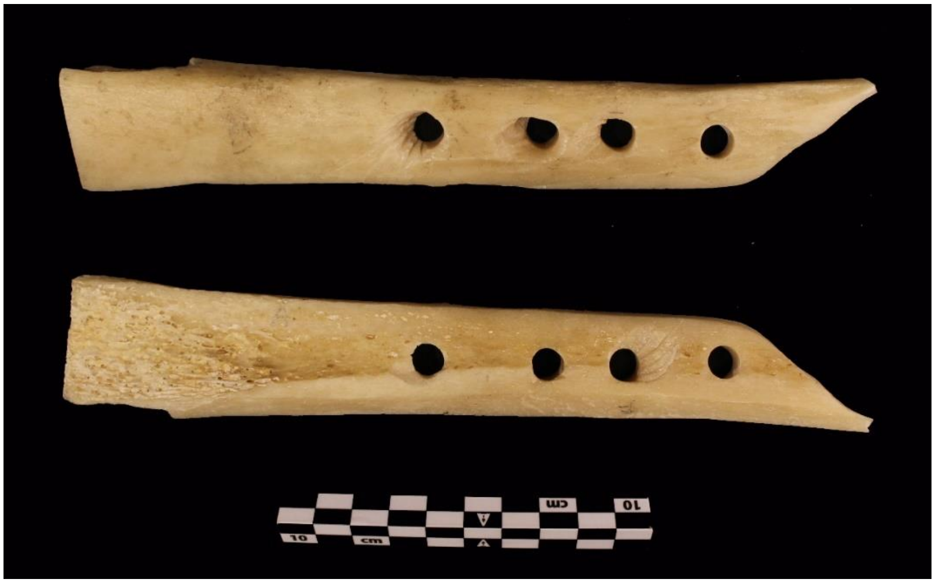

Fig. S11. Experimental piece in bone

Since the objective of the study did not concern the production process of the *Lochstab*, but the use of the artefact, metal tools were used for the perforation and grooves (which compensates for the more important hardness of bone). For making the grooves, we experienced no difference in terms of difficulty depending on whether the inner or outer groove was started with. It proved easier to keep the regularity

and symmetrical nature of the grooves when a stick (in wood or bone) was inserted in the hole to guide the grooving tool.

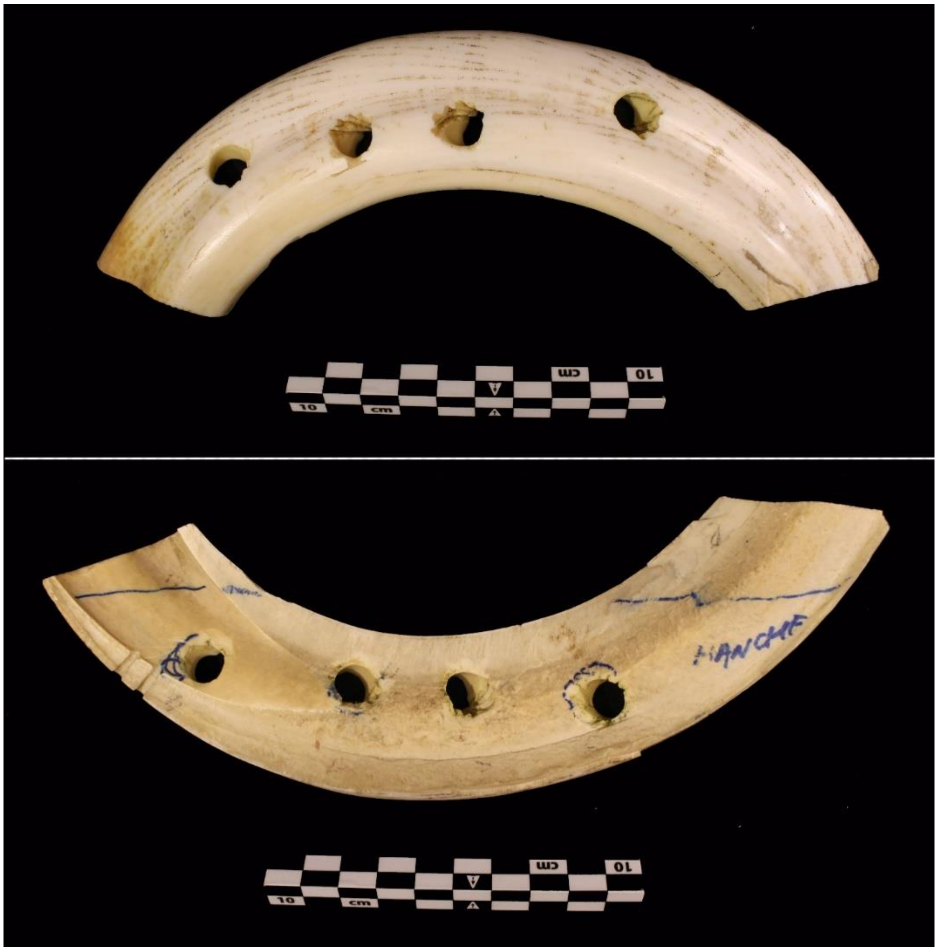

Fig. S12. Experimental piece in warthog tooth ivory

Finally, also a bronze version was produced on the basis of a silicone cast. This one was also used in the experiment.

Since we performed our experiments in 2016, another experimenter tried to reproduce the *Lochstab (50)* on the basis of the impressions we shared in the original field report *(7)* and in a conference presentation *(55)*. While the experiment is very interesting in terms of the production of the object as ivory could be used, the rope making concerned leather only, which is less relevant to the Aurignacian though successful results were also obtained (see *54* for more details).

Use of the reproduced Lochstabs

We tested different materials and explored the options if the holes functioned separately or together. The slightly differing sizes of the holes as well as the difference in degree of rotation (i.e. 180° or 360°) of the grooves between the holes is a point of attention here. We mainly focused on vegetal materials, but also explored the option of animal material. Worked materials consisted of tendons (deer), flax (*Linum*), hemp (*Cannabis*), reed mace or cat's tail (*Typha*), linden (*Tilia*), willow (*Salix*), and nettles (*Urtica*).

We first focused attention on the use of the artefact in breaking up stems to produce fibres. This only requires the use of a single hole. For materials like tendon, flax and hemp, we concluded that the *Lochstab* did not provide an added value to their processing and the tasks were easier to complete with a simple hammer stone. The reproduced *Lochstab* did not prove functional for breaking up dry tendons and for separating the fibres. The size of the holes limited the functionality of the tool, because the pieces of tendon were often too big to insert them easily into the holes. We also inserted flax twigs into the holes of the *Lochstab* and turned them around to try and break up the fibres. It works, but a hammer stone is much easier. A similar activity with hemp twigs did not work because hemp twigs were often too large to be inserted in the holes. The change in thickness between the lower and upper portion of the twigs also did not contribute to an easy breakup of the twigs with the artefact. Also for nettles, no added value of the artefact was noted. In the case of linden (*Tilia*), it is of course impossible to treat the bark with the *Lochstab* and the artefact was only used to combine the fibres by using more than one hole of the *Lochstab* (see below). By contrast, tests with reed mace (*Typha*) proved highly effective. The stems are a perfect fit for the holes and the grooves appear to help removing the outer harder surface of the *Typha*.

A second step in the manufacturing process concerns the combination of fibres and twisting them to produce a strand with a diameter corresponding to the size of the holes (these need to be counter-twisted later on to make rope). This process involves the actual use of the grooves. With linden fibres we tested their functionality. By using two holes at the same time, different lime tree fibres were pulled through the holes to check whether they would rotate automatically through their contact with the grooves. This did not happen. Subsequently, we tested whether, if the fibres were twisted by hand, the grooves within the holes had any effect on maintaining the torsion of the strands. This indeed proves to be the case to some extent. We also pulled bundles of *Typha* stems through the holes and the pressure of the bundle within the grooved hole combined with a gentle circular movement of the *Lochstab* allowed to easily produce a twisted strand. One hand (or an additional implement) of course needs to maintain the extremity of the strand after it has been pushed through the hole to maintain the twist. We found the artefact very effective for processing *Typha*. Strands with diameters of 7-8 mm can as such be produced that could be combined into a sturdy rope depending on the number of strands. By continuously feeding additional stems through the holes, long strands can be obtained.

The third step of combining strands into a rope requires the combined use of multiple holes. We first tested this with the two central holes only (360° rotation of grooves). Bundles of fibres twisted into strands and pulled through the object were secured or held at the other side of the artefact. Subsequently, a twist of the artefact in the opposite direction immediately combines the strands into a sturdy rope. A rope produced in this way would have a diameter of about 1.5 cm when two strands of about 8 and 7 mm (cf.

diameter of the holes) are used. A rope of such thickness is far more difficult to produce by hand and the use of the artefact finds its relevance there. The number of holes and thus strands that are combined determines the size and thickness of the rope. The grooves help maintaining the rotation of the strands, but not to the extent that these would be purely utilitarian in nature.

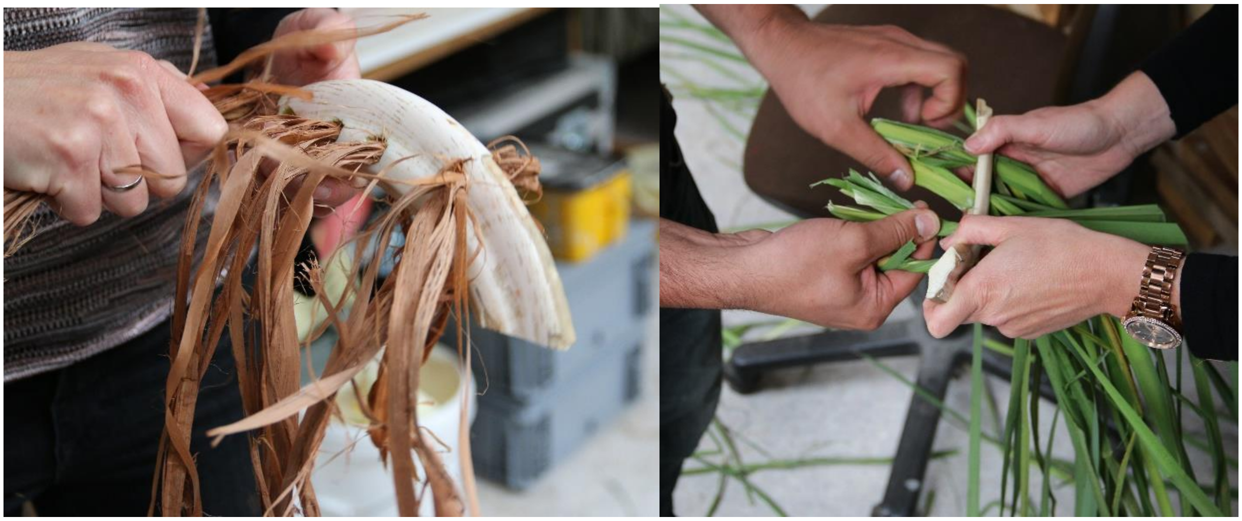

Fig. S13. Use of the ivory artefact for *Tilia* (left) and for *Typha* (right)

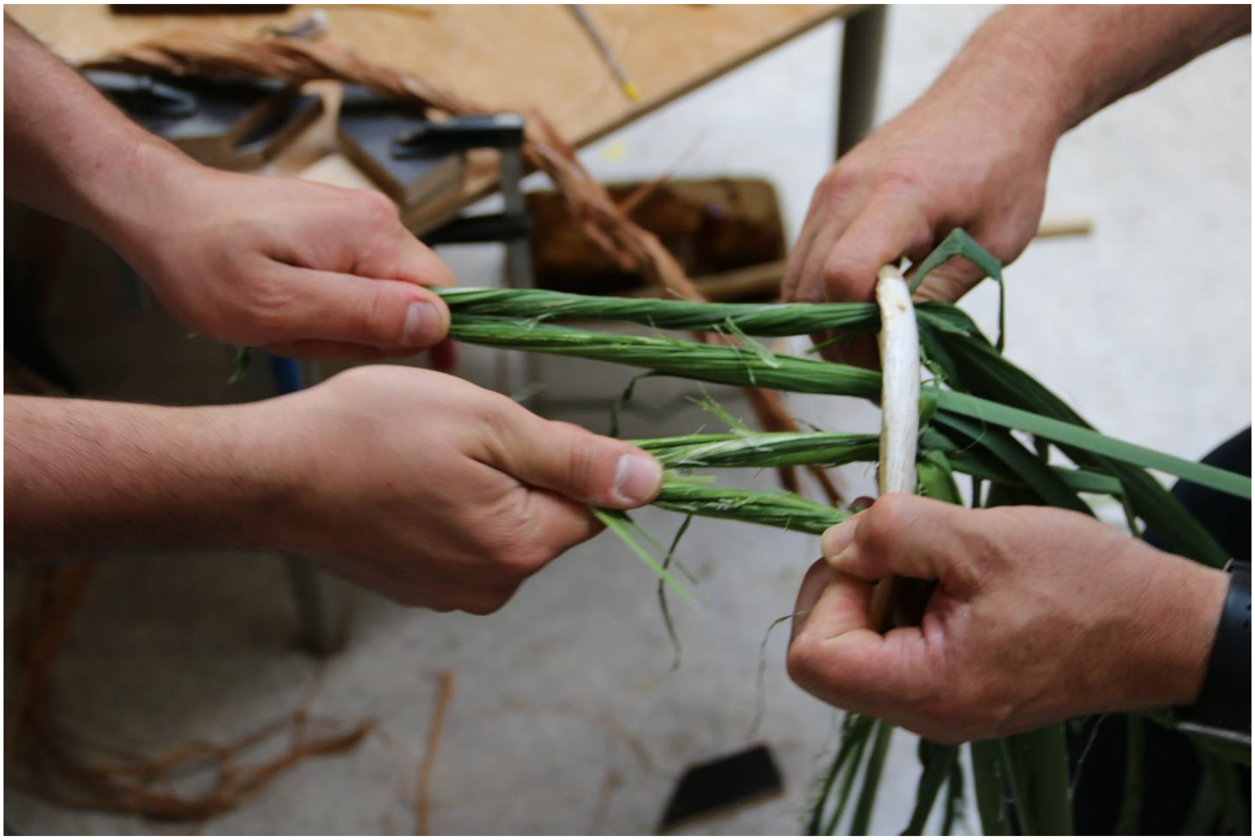

Fig. S14. Pulling the *Typha* through the holes and rotating fibres into strands by hand.

We subsequently tested the relevance of the tool when using the four holes together in a semi-automated protocol to manufacture rope according to what is known to have existed from at least the Egyptian pharaonic age onwards. The protocol was still widely spread in Medieval times. While hand-made rope production combines the twisting of fibres into a strand immediately followed by the combination of strands into a rope through a counter-twist, the process we refer to here works differently. First, the fibres are twisted (spun) into long strands and once this twist is complete over a long distance, they are combined into one sturdy rope. To maintain the torsion of the fibres, to keep the strands separate and to allow an “automatic” combination into a rope, a four-holed implement is used that is glided with a regular speed over the twisted strands. Behind the four-holed tool, the strands combine themselves automatically into a rope as a result of their twist. The *Lochstab* is a perfect fit for this process even if we are well aware that a much simpler tool could also be effective. In this kind of use, the grooves have no true functional relevance and would be decorative in nature (and through their rotation directly refer to a key characteristic of rope).

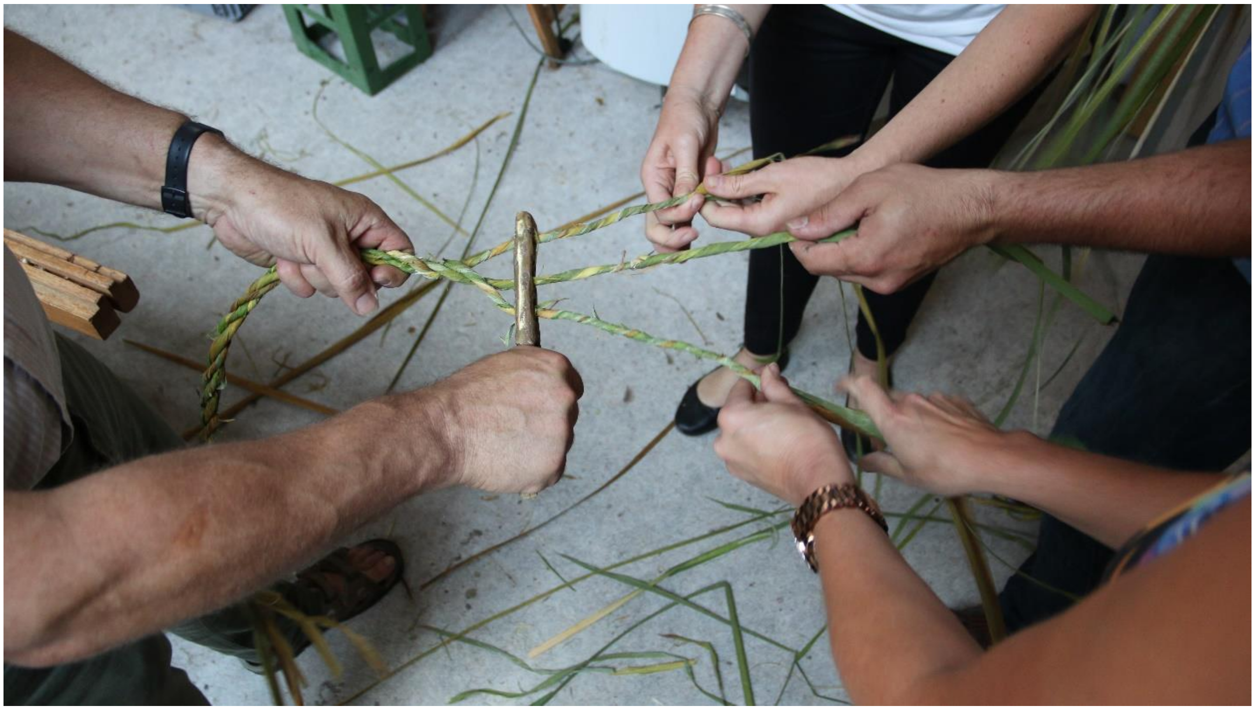

Fig. S15. Use of the bronze artefact to make rope from *Typha*.

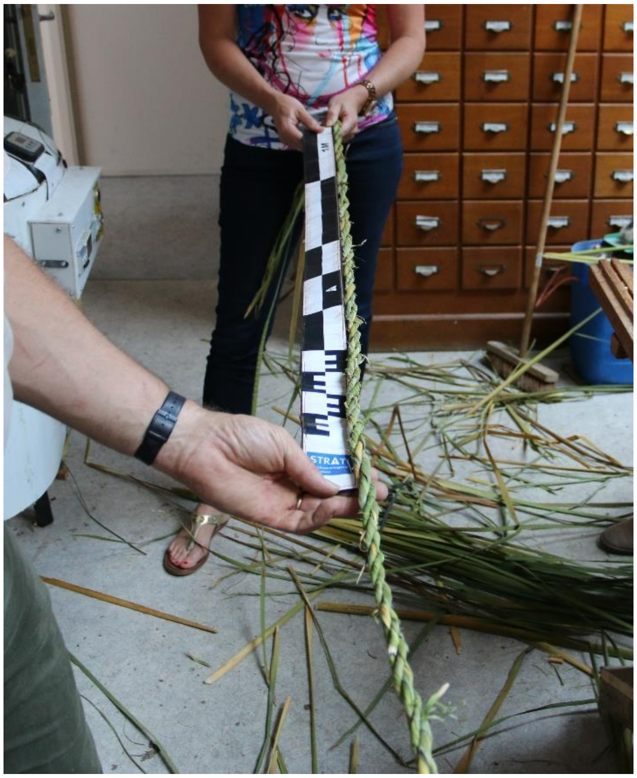

Fig. S16. Long ropes in *Typha* can as such be easily manufactured.

Functionality of the grooves

The spiral grooves within the holes of the *Lochstab* are carefully carved and very regular, which leads to the question of whether these grooves have a functional role or whether these are purely decorative. Other than advantages mentioned above, a possible functional role would be the twisting of the fibres within the strands. This was tested experimentally, but for the materials we tested, we found the grooves not to contribute significantly to the rotation of the fibres. The main reason is that leaves were principally used, which are wide and the grooves do not permit to twist them sufficiently. We expect the situation to be different when thin fibres were to be used. The grooves do however contribute to the maintenance of a twist provided manually and they proved very suitable for that purpose. We hypothesize that the grooves may contribute or permit the twisting of the fibres in the case of thin fibres, such as for instance animal or human hair, while the grooves maintain the twist provided manually in the case of other materials.

Conclusion

We conclude that the tool proves very effective to process *Typha* in view of rope manufacturing. The tool is effective throughout the whole process, from the break-up of the stems into fibres up to the combination of the twisted strands into a rope. Processing *Tilia* into rope proved similarly successful. While not tested to the same extent, also willow fibres (*Salix*) could be processed into a rope in a similar way. Other experiences performed since by others also proved successful in the use of comparable reproductions of the artefact for making rope though leather strips were used as basis *(50)*. The relevance of the Lochstab when compared to rope-making by hand is the possibility to produce sturdy ropes with thickness beyond what is easily manufactured by hand. In our experiment, we succeeded in producing a long and sturdy rope thanks to the use of the reproduced Lochstab.

Movie S1. Experiment in using a Lochstab in fiber processing

REFERENCES AND NOTES

1. L. Owen, *Distorting the Past: Gender and the Division of Labor in the European Upper Palaeolithic* (Kerns Verlag, 2005).

2. L. M. Hurcombe, *Perishable Material Culture in Prehistory: Investigating the Missing Majority* (Routledge, 2014).

3. O. Soffer, J. M. Adovasio, D. C. Hyland, The "Venus" figurines. *Curr. Anthropol.* **41**, 511–537 (2000).

4. C. Kilgore, E. Gonthier, Premières découvertes sur les techniques de fabrication de cordages à partir de rouets (Bâtons-percés). Évidences sur le mobilier et l'art pariétal du Paléolithique supérieur (Magdalénien). *Anthropologie* **118**, 347–381 (2014).

5. B. L. Hardy, M.-H. Moncel, C. Daujeard, P. Fernandes, Ph. Béarez, E. Desclaux, M.G. Chacon Navarro, S. Puaud, R. Gallotti, Impossible Neanderthals? Making string, throwing projectiles and catching small game during Marine Isotope Stage 4 (Abri du Maras, France). *Quat. Sci. Rev.* **82**, 23–40 (2013).

6. B. L. Hardy, M. H. Moncel, C. Kerfant, M. Lebon, L. Bellot-Gurlet, N. Mélard, Direct evidence of Neanderthal fibre technology and its cognitive and behavioral implications. *Sci. Rep.* **10**, 4889 (2020).

7. N. J. Conard, M. Malina, Außergewöhnliche neue Funde aus den aurignacienzeitlichen Schichten vom Hohle Fels bei Schelklingen. *Archäol. Ausgrab. Bad.-Württ.* **2015**, 62–66 (2016).

8. G. Bataille, N. J. Conard, Blade and bladelet production at Hohle Fels Cave, AH IV in the Swabian Jura and its importance for characterizing the technological variability of the Aurignacian in Central Europe. *PLOS ONE* **13**, *e0194097* (2018).

9. C. E. Miller, *A Tale of Two Swabian Caves—Geoarchaeological Investigations at Hohle Fels and Geißenklösterle* (Kerns Verlag Tübingen, 2017).

10. S. C. Münzel, N. J. Conard. Cave bear hunting in Hohle Fels Cave in the Ach Valley of the Swabian Jura. *Rev. Paléobiol.* **23**, 24–35 (2004).

11. J. Hahn, A. Scheer, Jungpaläolithische Feuerstellen in der Höhle Geißenklösterle, Blaubeuren, Alb-Donau-Kreis. *Archäol. Ausgrab. Bad.-Württ.* **1983**, 29–32 (1984).

12. J. Hahn, Die Geißenklösterle-Höhle im Achtal bei Blaubeuren I, in *Fundhorizontbildung und Besiedlung im Mittelpaläolithikum und im Aurignacien* (Theiss Verlag, 1988).

13. A. Glory, Nouvelle théorie d'utilisation des bâtons troués préhistoriques, in *Bulletin de la Société Historique et Archéologique du Périgord* (Perigueux, 1965), pp. 55–62.

14. T. Higham, L. Basell, R. Jacobi, R. Wood, C. Bronk Ramsey, N. J. Conard, Testing models for the beginnings of the Aurignacian and the advent of figurative art and music: The radiocarbon chronology of Geißenklösterle. *J. Hum. Evol.* **62**, 664–676 (2012).

15. G. Riek, *Die Eiszeitjägerstation am Vogelherd im Lonetal: Die Kulturen* (Akademische Verlagsbuchhandlung Franz F. Heine, 1934).

16. N. J. Conard, M. Bolus, Radiocarbon dating the late Middle Paleolithic and the Aurignacian of the Swabian Jura. *J. Hum. Evol.* **55**, 886–897 (2008).

17. A. Peltier, Bâtons percés, in *Fiches Typologiques de l'Industrie Osseuse Préhistorique, Cahier V Bâton Percés, Baguettes.* H. Camps-Fabrer, Ed. (CEDARC, 1992), pp. 7–70.

18. P. Noiret, Bâtons percés paléolithiques: Décor figuratif, schématique et géométrique. *Bull. Assoc. Sci. Liegeose Rech. Archeol.* **20**, 89–113 (1992).

19. C. Lucas, J. Galway-Witham, C.B. Stringer, S. M Bello, Investigating the use of Paleolithic perforated batons: New evidence from Gough's Cave (Somerset, UK). *Archaeol. Anthropol. Sci.* **11**, 5231–5255 (2019).

20. A. Schuler, Die Schussenquelle, in *Eine Freilandstation des Magdalénien in Oberschwaben* (Theiss Verlag, 1994).

21. J. Combier, Solutré, les fouilles de 1907 á 1925, in *Solutré.* M. Thoral, M. Riquet, J. Combier, Eds. (Imprimerie de Trévoux, 1955), pp. 93–220.

22. L. Didon, L'abri Blanchard des Roches. *Bull. Soc. Hist. Arch. Périgord* **38**, 246–261 (1911).

23. D. Peyrony, Les abris Lartet et du Poisson a Gorge d'Enfer (Dordogne). *Anthropologie* **42,** 241–268 (1932).

24. D. Peyrony, Le gisement Castanet, Vallon de Castelmerle, commune de Sergeac (Dordogne). Aurignacicen I et II. *Bull. Soc. Préhist. Française.* **32**, 418–443 (1935).

25. F. Delage, L'abri de la Souquette à Sergeac (Dordogne). *Bull. Soc. Hist. Arch. Périgord* **65**, 104–126 (1938).

26. J. Leclerc, D. Pradel, Un bâton orné du Magdalénien final de Loubressac, commune de Mazerolles (Vienne). *Bull. Soc. Préhistor. Française* **45**, 402–404 (1948).

27. E. Lartet, H. Christy, *Reliquiae Aquitanicae: The Archaeology and Paleontology of Perigord and the Adjoining Provinces of Southern France* (Baillere, 1967).

28. E. Peters, *Die Altsteinzeitliche Kulturstätte Petersfels* (Filser, 1930).

29. J. Riel-Salvatore, C. Gravel-Miguel, Upper Palaeolithic mortuary practices in Eurasia, in *The Oxford Handbook of the Archaeology of Death and Burial*, S. Tarlow, L.N. Stutz, Eds. (OUP, 2013), pp. 303–346.

30. P. Pettitt, *The Palaeolithic Origins of Human Burial* (Routledge, 2010).

31. A. Leroi-Gourhan, *Préhistoire de l'Art Occidental* (Mazenod, 1965).

32. K. Hardy, Prehistoric string theory. How twisted fibres helped to shape the world. *Antiquity* **82**, 271–280 (2008).

33. A. Revedin, B. Aranguren, R. Becattini, L. Longo, E. Marconi, M. Mariotti Lippi, N. Skakun, A. Sinitsyn, E. Spiridonova, J. Svoboda, Thirty thousand-year-old evidence of plant food processing, *Proc. Natl. Acad. Sci. U.S.A.* **107,** 18815–18819 (2010).

34. L. Hurcombe, Organics from inorganics: Using experimental archaeology as a research tool for studying perishable material culture. *World Archaeol.* **40**, 83–115 (2008).

35. S. Riehl, E. Marinova-Wolff, K. Deckers, M. Malina, N. J. Conard, Plant use and local vegetation patterns during the second half of the Late Pleistocene in southwestern Germany. *Archaeol. Anthropol. Sci.* **7**, 151–167 (2015).

36. N. J. Conard, M. Bolus. Radiocarbon dating the appearance of modern humans and the timing of cultural innovations in Europe: New results and new challenges. *J. Hum.* **44**, 331–371 (2003).

37. N. J. Conard, Cultural evolution in Africa and Eurasia during the Middle and Late Pleistocene, in *Handbook of Paleoanthropology,* W. Henke, I. Tattersall, Eds. (Springer, 2013).

38. M. L. Kochanski, *Northern Bush Craft* (Lone Pine Publishing, 1987).

39. E. Teeter, Techniques and terminology of rope-making in ancient Egypt. *JEA* **73**, 71–77 (1987).

40. D. Wescott, J. McPherson, Plant fiber preparation. *Bull. Primitive Technol.* **1** (2), 12–24 (1991).

41. M. Duhamel du Monceau, *L'art de la Corderie Perfectionné* (Connaissance et Mémoires Européennes, ed. 2, 1996).

42. A. L. West, *Aboriginal String Bags, Nets and Cordage, Revised Edition* (Museum Victoria, 2006).

43. L. Hurcombe, Organics from inorganics: Using experimental archaeology as a research tool for studying perishable material culture. *World Archaeol.*, **40**, 83–115 (2008).

44. C. Kilgore, E. Gonthier, Premières découvertes sur les techniques de fabrication de cordages à partir de rouets (Bâtons-percés). Évidences sur le mobilier et l'art pariétal du Paléolithique supérieur (Magdalénien), *Anthropologie* **118**, 347–381 (2014).

45. J. Hahn, Neue Beschleuniger-14C-Daten zum Jungpaläolithikumin Südwestdeutschland. *Eiszeitalter Gegenwart.* **45**, 86–92 (1995).

46. N. J. Conard, A female figurine from the basal Aurignacian of Hohle Fels Cave in southwestern Germany. *Nature* **459**, 248–252 (2009).

47. P. Reimer, W. Austin, E. Bard, A. Bayliss, P. Blackwell, C. Bronk Ramsey, M. Butzin, H. Cheng, R. Edwards, M. Friedrich, P. Grootes, T. Guilderson, I. Hajdas, T. Heaton, A. Hogg, K. Hughen, B. Kromer, S. Manning, R. Muscheler, J. Palmer, C. Pearson, J. van der Plicht, R. Reimer, D. Richards, E. Scott, J. Southon, C. Turney, L. Wacker, F. Adolphi, U. Büntgen, M. Capano, S. Fahrni, A. Fogtmann-Schulz, R. Friedrich, P. Köhler, S. Kudsk, F. Miyake, J. Olsen, F. Reinig, M. Sakamoto, A. Sookdeo, S. Talamo, The IntCal20 Northern Hemisphere radiocarbon age calibration curve (0–55 cal kBP). *Radiocarbon* **62**, 725–757 (2020).

48. C. Bronk Ramsey, Oxcal website (2023); https://c14.arch.ox.ac.uk/oxcal/OxCal.html#.

49. V. Rots, E. Hayes, D. Cnuts, C. Lepers, R. Fullagar. Making sense of residues on flaked stone artefacts: Learning from blind tests, *PLOS ONE* **11**, e0150437 (2016)

50. M. Legrand, R. Lam, M. Jensen-Fontaine, E. D. Salin, H. Man Chanac, 2004. Direct detection of mercury in single human hair strands by laser ablation inductively coupled plasma mass spectrometry (LA-ICP-MS). *J. Anal. At. Spectrom* **19**, 1287–1288.

51. B. L. Hardy, M. Bolus, N.J. Conard, Hammer or crescent wrench? Stone-tool form and function in the Aurignacian of southwest Germany. *J. Hum. Evol.* **54**, 648–662 (2008).

52. A. S. Wilson, H. I. Dodson, R. C. Janaway, A. M. Pollard, D. J. Tobin, Evaluating histological methods for assessing hair fibre degradation. *Archaeometry* **52**, 467–481 (2010).

53. V. Rots, 2010. *Prehension and Hafting Traces on Flint Tools. A Methodology* (Leuven Univ. Press, 2010).

54. T. Boucherat, Functional study of an archaeological tool of mammoth ivory with four spiral holes. *Mitt. Ges. Urgeschichte* **29**, 133–144 (2021).

55. N. J. Conard, V. Rots, Fiber technology, rope-making, textiles and the Lochstäbe from the Aurignacian of the Swabian Jura. *ESHE* **5**, 67 (2016).
